# Abiotic stress responses in forage crops and grasses: the role of secondary metabolites and biotechnological interventions

**DOI:** 10.3389/fpls.2025.1542519

**Published:** 2025-06-03

**Authors:** Udit Nandan Mishra, Jyoti Chauhan, Rajesh Kumar Singhal, Hirdayesh Anuragi, Prajjal Dey, Dalpat Lal, Saurabh Pandey, N.K. Gupta, Jajati Keshari Nayak, Aparna Tripathi, Mahendra Singh, Monika Yadav, Radha Sivarajan Sajeevan

**Affiliations:** ^1^ Faculty of Agriculture, Sri Sri University, Cuttack, India; ^2^ Department of Agriculture, Noida International University, Noida, India; ^3^ Division of Crop Improvement, ICAR-Indian Grassland and Fodder Research Institute, Jhansi, India; ^4^ Tree Improvement Research, ICAR-Central Agroforestry Research Institute, Jhansi, India; ^5^ Division of Basic Science & Humanities, Sher-e-Kashmir University of Agricultural Sciences and Technology, Srinagar, India; ^6^ College of Agriculture, Agriculture University, Jodhpur, Rajasthan, India; ^7^ Department of Molecular Biology and Biotechnology, Indira Gandhi Agricultural University, Raipur, India; ^8^ Department of Plant Physiology, Sri Karan Narendra Agriculture University, Jobner, India; ^9^ Department of Molecular Biology & Genetic Engineering, Govind Ballabh Pant University of Agriculture & Technology, Pantnagar, India; ^10^ Department of Agriculture, Jagan Nath University, Jaipur, India; ^11^ Department of Plant Protection Biology, Swedish University of Agricultural Sciences, Lomma, Sweden

**Keywords:** stress tolerance, secondary metabolites, growth and development, metabolic pathways, biotechnological interventions

## Abstract

Forage crops and grasses play crucial roles in global agriculture, serving as primary sources of livestock feed. However, various abiotic stresses, such as drought, salinity, extreme temperatures, and heavy metals, frequently challenge their productivity, quality, and resilience. In response to these stressors, plants activate defense mechanisms, including the production of secondary metabolites (SMs). This review exclusively examines the diverse impacts of abiotic stresses on forage crops and grasses’ physiological processes, growth, development, yield, and quality. We delve into the synthesis, types, and role of SMs in mediating stress responses, conferring adaptation and resilience to adverse environmental conditions in forage crops and grasses. Furthermore, we examine the regulatory mechanisms governing SM production in response to abiotic stress. This is crucial for developing strategies to enhance stress tolerance and improve forage productivity and quality. Finally, the review discusses emerging biotechnological interventions for improving forage crop performance under abiotic stress. Different omics technologies, gene editing, and pathway engineering offer promising avenues that enable the precise manipulation of key regulatory genes and metabolic pathways, with enhanced SM biosynthesis to engineer resilient crops tailored to specific environmental challenges. This review obtains a strong correlation of SMs with improving fodder and forage crop tolerance to varying degrees of stresses by regulating antioxidant activity, osmotic homeostasis, and membrane stability, ultimately enhancing plant viability, productivity, and quality under diverse stress conditions. Further, unraveling the intricate interplay between abiotic stresses, SMs, and biotechnological interventions is pivotal for advancing forage crop resilience and ensuring global food security amid changing environmental conditions.

## 1 Introduction

Developing nations like India are highly vulnerable to climate change primarily due to their agriculture-based economy compared to the developed nations (Chand et al., 2022a). Among the various adverse impacts of climate change, global warming stands out as the most prominent, given its diverse and far-reaching effects on all life forms, including agricultural crops, potentially threatening agricultural productivity and sustainability (IPCC, 2018; Gitz et al., 2016). The average global temperature has increased substantially throughout the 20th century due to climate change (Mondal et al., 2023). Furthermore, the escalating atmospheric CO_2_ concentration, driven by global warming, is anticipated to inevitably impact future global agricultural production by altering plant photosynthesis, primarily along with plant growth and transpiration rates (El Sabagh et al., 2021). Abiotic stresses, which encompass factors like temperature, salinity, and water deficit conditions, pose potential threats to plant health (Gupta et al., 2023).

Forage and fodder crops are vital components of agricultural systems, providing essential feed for livestock and contributing significantly to food security and economic stability globally. These crops support livestock production, which is crucial for the livelihoods of millions, particularly in developing countries where feed costs can account for up to 80% of total livestock production expenses (Kebede et al., 2024). These crops particularly support livestock production and enhance food security in developing countries by providing necessary nutrients and energy to livestock, which are vital for their growth, productivity, and overall health (Alam et al., 2010). By improving soil health, forage crops reduce the reliance on synthetic fertilizers, contributing to sustainable agricultural practices that are essential in the face of climate change and land degradation (Nour et al., 2023). However, climate changes impose various environmental stresses on forage crops, negatively impacting forage production (Singhal et al., 2023a). Climate change significantly alters pastures’ composition, growth, and development (Indu et al., 2023). The specific climatic condition requirements for cultivating forage, legumes, and perennial grasses necessitate crop scientists to breed climate-smart cultivars for the future. Even slight changes in climatic conditions can lead to considerable variations in green fodder productivity, depending upon the region’s climate, affecting changes in crop quality and quantity (Tyagi et al., 2023).

The adverse impacts of climate change, including rising temperatures and increased levels of atmospheric CO_2_, substantially reduce both the quantity of dry matter and the nutritional quality of forage crops (Singhal et al., 2023a). Climate change primarily impacts forage production by increasing CO_2_ levels, raising global temperatures, altering precipitation patterns, promoting weed growth, and intensifying the occurrence of extreme weather events (Sunil et al., 2020). Plants react to various abiotic and biotic stressful situations by modifying their genetic-level metabolism, leading to changes in metabolite synthesis, which is one of the prime strategies to strengthen their defense system (Jan et al., 2021a). As immobile organisms, plants adjust to stress by changing the expression levels of genes related to metabolite synthesis, which are released in response to different stressors (Samal et al., 2023). Plant secondary metabolites (PSMs) are specialized organic compounds generated through plant metabolism, serving as a defense against environmental stresses (Matthias and Kliebenstein, 2020). The levels of different SMs in plants heavily rely on their growth stage and environmental conditions, which, in turn, can significantly impact the metabolic pathways involved in synthesizing and accumulating these compounds.

The synthesis of SMs in plants can occur as either constitutive or induced. Certain SMs are consistently produced and are termed constitutive SMs. However, SM synthesis imposes a substantial metabolic burden on the host plant, so many of these compounds are not synthesized in significant amounts until after certain external stimuli, such as insect feeding. Such SMs are referred to as induced SMs (Freeman and Beattie, 2008). Among the various SM categories, terpenoids represent the largest and the most diverse category, found across all plant species (McGarvey and Croteau, 1995; Yazaki et al., 2017; Zeng et al., 2022). Isoprene (C_5_H_8_) stands as the simplest terpenoid, existing as a volatile gas generated during leaf photosynthesis. Terpenoids are classified based on the number of isoprene units in their structures, with monoterpenoids composed of two units, sesquiterpenoids of three units, diterpenoids of four units, and triterpenoids of six units. Phenolics constitute a significant category of SMs, encompassing diverse defense-related compounds like flavonoids, anthocyanins, phytoalexins, tannins, lignin, and furanocoumarins (Freeman and Beattie, 2008).

The functional roles of SMs vary significantly between non-forage and forage crops. For instance, phenolic compounds in fruits like apples and grapes enhance antioxidant activity, extending shelf life and nutritional value. In contrast, forage crops like alfalfa utilize phenolics to reduce protein degradation during digestion, enhancing nutrient absorption in livestock. Similarly, alkaloids in coffee and tobacco act as natural pest deterrents, whereas in forage grasses like perennial ryegrass, they minimize pest-related damage and reduce dependency on chemical pesticides. Saponins in crops like quinoa contribute to pest resistance and health benefits, but in forage legumes like clover, they reduce methane emissions in ruminants, enhancing environmental sustainability. Flavonoids in tomatoes and soybeans provide UV protection and improve flavor, whereas in forage legumes like red clover, they influence animal fertility and protect plants against oxidative stress. Likewise, terpenoids enhance the aroma and flavor in commercial crops like oranges and peppermint but serve as barriers for pests in certain tropical forage grasses, aiding their survival under challenging conditions.

Abiotic stresses such as drought, salinity, and cold significantly impact SM production in plants. Anthocyanins tend to accumulate under drought and cold conditions, and tissues containing higher anthocyanins display significant stress resistance (Solíz-Guerrero et al., 2002). Osmotic stress in plants is induced by salt stress, resulting in alterations in the accumulation of specific SMs. Studies indicate that anthocyanin levels tend to elevate when plants are exposed to salt stress (Parida and Das, 2005). Exposure to cold stress enhances the production of phenolics, leading to their subsequent integration into the cell wall, in the form of either suberin or lignin (Griffith and Yaish, 2004). A number of previous studies have focused on major crops and found that the production of SM is crucial for enhancing stress tolerance capacity. However, forage crops are quite potential crops for climate resilience, but the role of SMs has not been studied thoroughly. Unlike previously published reviews, we focused primarily on the mechanistic role of SM and their production as a means of abiotic stress defense through the modulation of physiological, biochemical, and molecular mechanisms through biotechnological interventions. Further, this review provides insight into the application of advanced biotechnological techniques, including genome editing and alteration of bioengineering pathways for the development of climate-smart forage crops.

## 2 Impact of abiotic stresses on plant growth and development

A prominent consequence of global climate change is the continual curtailment in the availability and productivity of arable land. Simultaneously with climate change, abiotic stresses significantly limit crop productivity and quality (Shabbir et al., 2022; Chand et al., 2022a). Abiotic stresses, individual or combined, substantially influence the crucial processes, signaling networks, and gene functions of crop plants’ influence on growth and development (Gupta et al., 2023). A recent report estimated that unfortunate environmental circumstances negatively affect crop survival, growth, and biomass production and threaten global food and nutritional security, as well as environmental safety (Gitz et al., 2016). Like other crops, forage crops are more concerned with multiple abiotic stresses and are subjected to more significant losses in yield and productivity, affecting livestock nutrition and productivity (Indu et al., 2023). The effect of major abiotic stress on plant processes, as well as growth and development, is discussed thoroughly.

### 2.1 Drought stress

Drought stress is also well documented for its inhibitory action on photosynthesis, primarily due to stomatal closure, reducing CO_2_ availability, and damage to the photosynthetic apparatus’s major components, such as Photosystem I (PSI) and Photosystem II (PSII). Damage leads to higher photoinhibition, modification of photosynthetic pigment composition, and disruption of chloroplast biochemistry (Kong et al., 2015). At the vegetative phase, drought remarkably diminished the concentrations of photosynthetic pigments (chlorophyll and carotenoids) and irreversibly damaged Photosystem I and II at high-stress conditions (Chauhan et al., 2023a). In limited water, turgor pressure loss in plants induces stomatal closure as a physiological adjustment to reduce transpiration and conserve water in cells (Fariaszewska et al., 2020). Water deficits diminish the availability and uptake of mineral nutrients by modulating root structure architecture and disrupt water and nutrient transport in the xylem and phloem vessels (Indu et al., 2021). In forage grasses, drought stress is also linked with reduced variable fluorescence to maximum fluorescence ratio (Fv/Fm), a widely accepted indicator of photosynthetic performance. Low Fv/Fm reflects photoinhibition, compromised photosynthetic capability, and general downregulation of photosynthesis (Fariaszewska et al., 2020). Drought has also been shown to increase crude protein content, possibly due to retarded plant maturity or enhanced soil nitrogen availability concerning water content under water deficit conditions (Staniak, 2013). To ensure cellular homeostasis during drought stress, plants deploy osmotic adjustment mechanisms, building up osmolytes like water-soluble carbohydrates and proline. These substances regulate osmotic potential, stabilize cellular turgidity, and compensate for the negative impacts of dehydration (DaCosta and Huang, 2006; Fariaszewska et al., 2020). This complex physiological and biochemical response reflects the sophisticated strategies adopted by plants to compensate for water deficit and maintain metabolic function during drought.

The production of SMs, particularly phenolic compounds, is widely reported in drought-stressed plants and is considered a crucial adaptive mechanism to offset the adverse effects of water deficiency (Jaafar et al., 2012). Phenolic compounds, being highly radical scavenging, are key to mitigating oxidative damage and protecting the photosynthetic machinery under conditions of drought and oxidative stress (Nakabayashi et al., 2014). Photosynthesis is a dual-function molecule in plant metabolism, which generates energy in the form of ATP and NADPH and provides carbon skeletons required for the biosynthesis of primary and SMs. Correlation studies of various past research have revealed a significant negative correlation between photosynthetic activity and flavonoid production in drought stress conditions (Jan et al. 2021b). This implies that photosynthetic downregulation under stress causes plants to rechannel limited carbon resources to the biosynthesis of SMs, specifically flavonoids, through the shikimic acid pathway. The metabolic pathway is key in the biosynthesis of phenolic compounds such as flavonoids that reinforce plant defense against abiotic stress agents. The reported negative correlation results from a trade-off strategy whereby compromised photosynthetic efficiency forces plants to utilize the scarce carbon resources to stress adaptation at the cost of growth and biomass acquisition. With the reduction in photosynthetic carbon assimilation, secondary metabolism becomes activated to reinforce the protective functions of the plant. Transcriptomic and metabolomic analyses of Dinanath grass revealed that drought stress triggers the upregulation of flavonoid biosynthetic genes and the accumulation of flavonoids (Puttamadanayaka et al., 2024). Flavonoids accumulate specifically due to their antioxidant and stress-relieving activity and help in alleviating drought-stimulated cellular damage. The enhanced synthesis of SMs under restricted photosynthesis is then accounted for in terms of intensified flux through the shikimic acid route, a noted metabolic response during abiotic stressful conditions such as water stress (Rahmat et al., 2008).

Drought compels plants to trigger complex changes in their genes and chemicals. This helps them to produce more SMs, which are critical for coping with stress due to lack of water and sustaining stability in their cells. Secondary metabolites like flavonoids, phenolic acids, terpenes, and carotenoids are natural antioxidants that assist in the removal of harmful reactive oxygen species (ROS) formed during drought (Khatun et al., 2021). The biosynthesis of these metabolites is controlled predominantly at the gene level by specific proteins called drought-responsive transcription factors (TFs), which are members of families like MYB, bHLH, WRKY, NAC, and DREB. These TFs interact with specific sequences of the DNA in the genes producing compounds like phenylalanine ammonia lyase (PAL), chalcone synthase (CHS), and terpene synthase (TPS) and, through this, the flow of chemicals via key routes like the phenylpropanoid, mevalonate, and methylerythritol phosphate (MEP) pathways (Ghasemi et al., 2023).

### 2.2 Salinity stress

Salinity impacts plants primarily through osmotic stress, which results in lower water absorption and consequently impacts turgor pressure and nutrient uptake. Earlier studies have proven that elevated levels of salinity result in lower germination and seedling development at the beginning. This is mainly attributed to osmotic stress, which restricts water uptake and results in improved delay in germination or lower seedling biomass (Khanduri et al., 2021; Dwivedi et al., 2022). Plants cannot absorb water as soil salinity increases, causing them to lower transpiration by reducing stomatal conductance (Lu et al., 2021). Increased salinity levels may contribute to ion toxicity, where an excessive build-up of sodium (Na^+^) and chloride (Cl^−^) ions disrupts the cellular balance and hence impacts crucial physiological processes such as nutrient uptake and metabolic processes (Lu et al., 2021). Such ion imbalance may impede the transport of necessary nutrients, aggravating nutritional deficiency and ultimately inhibiting plant growth and development.

The inhibited water absorption and ionic imbalance minimized cell enlargement, resulting in dwarfed growth. Lower turgor pressure and inhibited photosynthetic efficiency restricted leaf growth, hence minimizing leaf area. This reduction affects light capture and overall plant productivity. As a result of the ineffectiveness of photosystems in capturing adequate light, photosynthetic reduction contributed to biomass reduction due to inhibited photosynthesis, nutrient deficiencies, and low vegetative growth (Sharavdorj et al., 2021; Tokas et al., 2021). In severe cases of salinity stress, programmed cell death may also occur (El Sabagh et al., 2021). The salinity stress triggers an overproduction of ROS owing to the disruption of electron transport chains (ETCs) in chloroplasts and mitochondria (Bell and Smith, 2021; Saha et al., 2022). In saline environments, plants experience increased levels of ROS, including singlet oxygen, hydroxyl radicals, superoxide radicals, and hydrogen peroxide. These highly reactive molecules are capable of inflicting extensive damage on cellular components, including proteins, lipids, and DNA, thereby initiating oxidative stress (Bell and Smith, 2021; Hou et al., 2022). Upon oxidative stress, plants initiate antioxidant defense systems that include the upregulation of antioxidant enzymes (Atta et al., 2023). Augmented antioxidant activity has been correlated with enhanced physiological traits in salt-resistant crops, thereby supporting better development and yield in salinity stress conditions. Plants grown under salinity stress often exhibit significantly higher levels of certain secondary plant products than in normal cultivated conditions. Secondary metabolites in plants are usually involved in the modulation of antioxidant pathway genes such as superoxide dismutase (SOD), catalase (CAT), peroxidases, PAL, and glutathione-*S*-transferase (GSTs), which serve in plant defense against salinity stress. The accumulation of polyphenols is an excellent example of a plant’s response to salinity stress, which is attributed to SM accumulation (Singhal et al., 2021). The tolerant genotypes of forage crops were reported with significantly higher amounts of SMs like phenols, saponins, flavonoids, carotenoids, and lignin under superposition of osmotic and ionic stress to cope with the secondary oxidative stress.

Salinity detection by membrane sensors begins to initiate signaling in the cells. This involves pathways referred to as salt overly sensitive (SOS) and modules referred to as mitogen-activated protein kinase (MAPK), which regulate stress response genes (Zhu, 2016). At the center of this regulation are TFs such as MYB, bHLH, NAC, WRKY, and DREB. These TFs directly impact the promoters of genes that produce SMs, such as PAL, CHS, 4CL, and TPS (Meraj et al., 2020; Bageel et al., 2022). These TFs enhance the flow of metabolites through pathways generating phenylpropanoid, terpenoid, and carotenoid, which allows the plant to protect against oxidative and ionic stress.

### 2.3 Temperature stress

High temperatures can damage photosynthetic machinery and reduce the activity of vital enzymes like ATP synthases. High temperature, especially, enhances photorespiration due to the reduced affinity of the enzyme RuBisCO to carbon dioxide, leading to reduced carbon fixation. A plant defense mechanism, stomatal closure, to conserve water also results in reduced photosynthesis and biomass accumulation under conditions of heat stress (Nour et al., 2023). Temperature stress causes oxidative stress by disturbing redox homeostasis and elevating the production of free radicals. These can alter gene expression, inactivate enzymes, and lead to membrane degradation, which is also supported by elevated malonaldehyde activity in various studies (Ghalkhani et al., 2023). Temperature stress includes chilling (0°C–15°C), freezing (<0°C) (low temperature), and heat stress (27°C–40°C) (high temperature) (Bhat et al., 2022). Cellular membrane integrity is a fundamental component directly associated with plants sensitive to temperature stress. Various physicochemical changes occur within plant cell membranes to ensure membrane stability and function, enabling plants to survive temperature changes. Some of these changes include remodeling lipids to maintain fluidity, regulating ion channels to balance Na^+^ and K^+^, and modifying proteins like aquaporins and transporters to optimize water and ion use. When crops and plants are exposed to low temperatures, the lipids within cell membranes undergo a transition from a liquid to a solid state. This transition is primarily affected by the ratio of unsaturated fatty acids present. Recent studies have indicated that plants activate enzymatic and non-enzymatic antioxidant defense mechanisms to adapt to temperature fluctuations. To mitigate low-temperature stress, plants synthesize various osmolytes and osmoprotectants, including lipids, proline, glycine betaine, and sugars, which aid in reducing membrane damage (Ritonga and Chen, 2020). Heat stress impacts the biochemical and physiological functions of the plant by altering molecular mechanisms. Higher temperatures beyond the threshold level delay seed germination and affect the crop stand establishment of the crop (Mondal et al., 2023; Sharavdorj et al., 2021). Heat not only inflicts cellular damage on plants but also disrupts numerous intricate processes and structures, ultimately leading to plant death. Proteins can also be inactivated by high temperatures, leading to deactivation in enzyme function and an upsurge in the production of ROS and active oxygen species (AOS) (Zhao et al., 2012). The reproductive stage is severely affected by high temperatures, as it reduces pollen germination, fertilization, and grain development, which ultimately causes huge yield penalties in forage crops (Niazi et al., 2020). Additionally, the quality of the produce suffers from heat stress, leading to reductions in starch and protein contents.

### 2.4 Heavy metal stress

Plants can absorb heavy metals (HMs) through their surfaces, whether submerged underwater or above ground. Exposure to plants beyond the threshold level of heavy metals triggers a wide range of physio-biochemical and metabolic alterations. A few crops and forage species exhibit varying degrees of HM tolerance and adapted to contaminated soils. For example, *Medicago sativa* and *Lolium perenne* are widely recognized for their ability to accumulate cadmium, lead, and zinc while also maintaining high biomass production, which is crucial for forage applications (Chaudhry et al., 1998; Rai, 2008; Thayalkumaran et al., 2003; Meng et al., 2011). *Trifolium repens* and *Pennisetum purpureum* demonstrate tolerance to HMs and contribute to phytoremediation strategies due to their rapid growth and adaptability to diverse soils (Liu et al., 2015; Yun and Ali, 2019; Lin et al., 2021; Rahman et al., 2024). Once the HMs accumulate beyond the threshold levels, it has significant effects on plants’ growth and development. It can often lead to a reduction in growth rates, manifested by symptoms such as leaf chlorosis, necrosis, decreased turgor, seed germination rate, and impaired photosynthetic apparatus. All these responses are related to ultrastructural, biochemical, and molecular modification in plant systems caused by HMs (Singhal et al., 2023b). Heavy metal toxicity often leads to the excessive buildup of ROS and methylglyoxal (MG) at the cellular level (Zhang H. et al., 2021). Both ROS and MG can induce lipid peroxidation, protein oxidation, enzyme inactivation, and DNA damage and potentially interact with other critical components of plant cells. It also involves the disturbance of protein structural building blocks due to the formation of bonds between HMs and sulfhydryl groups, thereby interfering with the functional groups of essential cellular components. Heavy metal stress can result in reduced crop productivity, seed germination, accumulation, and re-mobilization of seed metabolites during plant growth (Keyster et al., 2020). Both germination and photosynthesis lead to deleterious effects on various plant physiological processes. Ultimately, these effects can disrupt normal growth processes and cause senescence and even plant death (Kchaou et al., 2020).

Many studies have proven that seed germination in fodder crops is negatively influenced by the presence of heavy metals. It was found that lead ions interfere with seed enzymes involved in the hydrolysis and transport of essential food reserves like carbohydrates and proteins, which are essential for metabolic processes and embryonic growth. This interference presents a viable reason for germination inhibition in many studies (Singh et al., 2011; Jócsák et al., 2022). In addition, the inhibitory action on germination can be attributed to changes in the permeability properties of cell membranes. It could also be due to the reduction in viability caused by the decrease in energy production by the embryo.

Excess zinc causes interference with plant homeostasis, leading to the inhibition of growth, leaf chlorosis, disrupted chlorophyll biosynthesis, and lowered photosynthetic rates, thereby interfering with cellular signaling and seed germination (Yahaghi et al., 2019). Nickel is a cytosolic metalloenzyme that catalyzes urea hydrolysis in support of nitrogen assimilation. Furthermore, excess exposure to nickel was also found to be associated with the upregulation of peroxidase glutathione-*S*-transferase activities and malondialdehyde content, which are oxidative stress and lipid peroxidation indicators (Myrach et al., 2017).

## 3 Secondary metabolites: synthesis, types, and role in plant growth and development

SMs are structurally diverse organic compounds synthesized by plants, fungi, and microorganisms. Unlike primary metabolites, which are indispensable for fundamental growth, energy metabolism, and biomass accumulation, SMs primarily mediate ecological interactions and enhance stress adaptation (Simpson et al., 2018). In forage and grass species, SMs such as phenolics, flavonoids, and terpenoids play pivotal roles in modulating abiotic and biotic stress responses. They integrate with key stress signaling pathways, particularly those mediated by abscisic acid (ABA), jasmonic acid (JA), and salicylic acid (SA), functioning as signaling enhancers, ROS scavengers, and membrane stabilizers (Austen et al., 2019). These biochemical activities enable plants to fine-tune stomatal regulation, osmotic adjustment, and pathogen defense under stressors like drought and herbivory. In contrast, primary metabolites support routine physiological processes and are essential under optimal growth conditions. However, under stress conditions, SMs become more crucial for survival. This shift, though, involves significant trade-offs: the biosynthesis of SMs requires high carbon and nitrogen input, potentially reducing forage yield and digestibility, especially in nutrient-poor environments (Bhatla and Lal, 2023; Reshi et al., 2023). SMs often exhibit species-specific ecological roles such as allelopathy, interplant communication, and defense, well documented in phenolics and alkaloid compounds (Chauhan et al., 2017; Divekar et al., 2022; Chauhan et al., 2023b; Mishra et al., 2023). Their taxonomic specificity suggests evolutionary adaptation tailored to environmental pressures (refer to **Table 1**). Beyond ecological significance, SMs often exhibit bioactivity that can be exploited for medicinal, agricultural, or industrial purposes, making them valuable resources for human use (Twaij and Hasan, 2022).

**Table 1:** Gene and their encoded products (SMs) expressed under various abiotic stress conditions in forage/grass crops.

Forage crop/grass	abiotic stress condition	Gene expressed upon stress	Secondary metabolite responsible	Defense mechanism	Reference
*Beta vulgaris*	Salinity	–	Alkaloid (berberine), phenolics (flavonoid)	Oxidation inhibition, free radical scavenging	Liu et al. (2020)
*B. vulgaris*	Salinity	*BvGR*	*S*-Containing compound (glutathione)	Removal of ROS	Bor et al. (2003)
*B. vulgaris*	Salinity	–	Terpenoid (ABA)	ABA-mediated activation of downstream effectors *viz.*, TFs and ion channels, osmoprotectants, and stress-responsive genes	Cui et al. (2022)
*Brassica napus*	Light	*BnCRY1*, *BnCRY2*	Phenolics (flavonoid)	Increased anthocyanin level leads to photomorphogenesis	Sharma et al. (2014)
*B. napus*	Light	*BnCHS*, *BnCHI*, *BnFLS*, *BnF3′H*, *BnF3H*, *BnDFR*, *BnANS*, *BnUFGT*, *BnPAP2*, *BnGL3*	Phenolics (anthocyanin)	Direct upregulation of anthocyanin biosynthetic pathway gene; indirect upregulation of anthocyanin production mediated by MYB and bHLH type TF; photoprotection and ROS scavenging	Luo et al. (2021)
*B. napus*	UV-B irradiation	*CHS*, *CHI*, *F3H*, *F3′H*, and *DFR*	Phenolics (flavonoid)	Increase the levels of antioxidants and reduce photo-oxidative damage at leaf epidermis by inhibiting the penetration of UV-B irradiation into the inner photosynthetic layers	Lee et al. (2021)
*B. napus*	Nutrient stress (high nitrogen)	*ANS* and *DFR-like1*	Phenolics (anthocyanin)	Photoprotection through osmolyte accumulation and prevention of photooxidation damage	Koeslin-Findeklee et al. (2015)
*B. napus*	Cold	*FLS*	Phenolics (flavonoid)	Upregulation of *trans*-cinnamate 4-monooxygenase, flavonol synthase, shikimate *O*-hydroxycinnamoyltransferase followed by ROS scavenging through flavonoid	Mi et al. (2021)
*B. napus*	Drought	*CHS*, *CHI*, *F3′H*, *DFR*, *ANS*, and *UGT79B1*	Phenolics (anthocyanin)	ROS scavenging, osmotic balance	Chen W. et al. (2022)
*Brassica rapa*	Salinity, drought, cold	*CCD*, *NCED*	Terpenoid (carotenoid)	ABA, apocarotenoid, and strigolactone mediated protection against stress by catalyzing the conversion of 9′-*cis*-β-carotene to 9-*cis*-β-apo-10′-carotenal followed by 9-*cis*-β-apo-10′-carotenal to carlactone 9′-*cis*-β-carotene to 9′-*cis*-β-carotene + β-ionone to apocarotenoid ABA–aldehyde to ABA	Kim et al. (2016)
*B. rapa*	Cold	*PAL, C4H, 4CL, CHS, CHI, F3H, F3′H, and FLS, DFR, ANS, LDOX, UFGT*	Phenolics (anthocyanin)	Activation of phenylpropanoid and flavonoid biosynthetic pathway thereby ROS scavenging	Dai et al. (2022)
*B. rapa*	UV	*BrPAL, BrC4H, Br4CL, BrCHS, BrCHI, BrF3H, BrF3′H, and BrFLS, BrDFR, BrANS, BrLDOX, BrUFGT*	Phenolics (flavonoid)	Act as an antioxidant and also absorbs incoming UV-B rays	Hao et al. (2022)
*Hordeum vulgare*	Salinity	*Hds1*	Terpenoids	Upregulation of hydroxy methyl butenyl 4-diphosphate synthase, an enzyme for the penultimate step of MEP pathway, leads to increased structural sterol biosynthesis, thereby altering the permeability and fluidity of root plasma Membrane (PM)	Witzel et al. (2014)
*H. vulgare*	Nutrient stress (low potassium)	–	Not clear	Increased PAL activity leads to increased accumulation of *trans*-cinnamic acid, leading to the synthesis of phenylpropanoid skeletons *viz.*, flavonoids, lignin, and alkaloids	Zeng et al. (2018)
*H. vulgare*	Drought + salinity	*GST1*, *PPO*, *SKDH*, *PAL*, *CAD*, and *chi2*	Phenol, flavonoid	Increased callose content, sucrose synthase, sucrose phosphate synthase, and acid invertase activity	Ahmed et al. (2015)
*H. vulgare*	Elevated CO_2_ + O_3_ level	–	Phenolics (phenylpropanoids and phenolamides)	Act as antioxidants	Mikkelsen et al. (2015)
*H. vulgare*	Heavy metal (Cd)	–	Phenolics	Act as antioxidant; reduce lipid peroxidation and counteract ROS generation	Večeřová et al. (2016)
*Saccharum* sp*ontaneum*	Cold	*LDOX*	Phenolics (anthocyanin)	Formation of pro-anthocyanin leads to ROS quenching	Selvarajan et al. (2018)
*Sorghum bicolor*	Drought	*CCD*	Terpenoid (carotenoid)	Tolerance against ROS generated during drought stress in coordination with sulfur-containing SM glutathione	Woldesemayat and Ntwasa (2018)
*S. bicolor*	Light	*SbPAL, SbC4H, Sb4CL, SbCHS, SbCHI SbF3H, SbFNSII, SbF3′H, SbFNR, SbDFR, SbANS*	Phenolics (flavonoid)	Detoxification of damaging free radicals	Fedenia et al. (2020)
*Zea mays*	Drought	*TPS6/11, AN2*	Terpenoids (zealexins and kauralexins)	Maintains membrane integrity by quenching ROS through systemic ABA and JA signaling	Vaughan et al. (2015)
*Avena sativa*	High altitude	–	Terpenoid (carotenoid)	Photo-protection of photosynthetic pigment system through upregulation of zeta-carotene isomerase, zeaxanthin epoxidase, and xanthoxin dehydrogenase leading to optimization of chlorophyll to carotenoid pigment proportion	Jinqiu et al. (2021)
*Pennisetum glaucum*	Drought	–	Terpenoids, phenolics (flavonoid)	Strictosidine synthase 1 and flavanone 3-dioxygenase promote biosynthesis of terpenes and flavonoids, respectively. Subsequent formation of xanthophyll, anthocyanin, antheraxanthin, and zeaxanthin helps in dissipation of high-intensity light through anti-photooxidative effect	Shivhare et al. (2020)

SM, secondary metabolite; ROS, reactive oxygen species; TFs, transcription factors; PAL, phenylalanine ammonia lyase.

Chemically, SMs are distinguished by complex biosynthetic modifications such as methylation, glycosylation, and hydroxylation, resulting in elaborate structures than primary metabolites. A comparative analysis of SM pathways, including curated gene orthologs, is provided in **Supplementary Table 1** (https://pmn.plantcyc.org/group?id=pmn.plantcyc.org-7711-3920509542). Three primary categories of plant metabolites are distinguished by their biosynthetic pathways (Agostini-Costa et al., 2012; Twaij and Hasan, 2022), as follows:

- terpenoids (composed mainly of carbon and hydrogen),
- phenolic groups (consisting of simple sugars and benzene rings), and
- nitrogen-containing compounds.

### 3.1 Biosynthesis pathway of secondary metabolites

**Figure 1:**
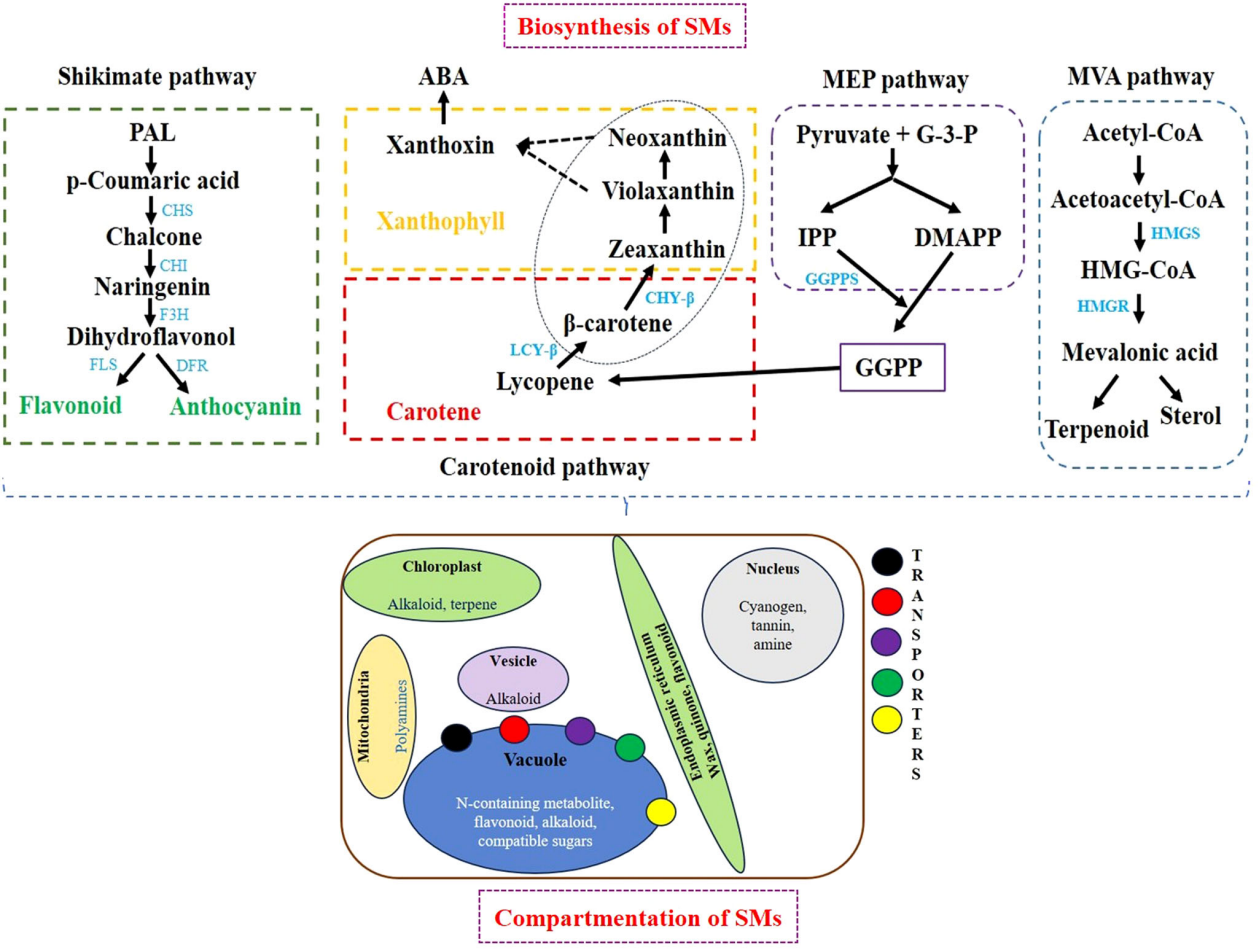
Biosynthesis and compartmentalization of SMs. SMs, secondary metabolites.

Plant SMs are categorized into three distinct chemical groups: terpenes, phenolics, and nitrogen-containing compounds. A comparative SM biosynthesis pathway and compartmentation are illustrated in **Figure 1**. The primary pathway involved is the shikimic acid pathway, a crucial process within chloroplasts that provides the precursors for phenylpropanoid compounds. Phenolic compounds are synthesized via the malonate pathway. In plant cells, chloroplasts convert carbon dioxide from the atmosphere into glyceraldehyde-3-phosphate through photosynthesis. The products of these reactions accumulate as carbohydrates, which are subsequently broken down by the glycolysis process in the cytosol during the mevalonate metabolism occurring in the cytosol and mitochondria of eukaryotic cells. The mevalonate pathway utilizes acetyl-CoA and glucose to synthesize cholesterol and fatty acids through a series of enzymatic steps and the tricarboxylic acid (TCA) cycle in the cytoplasm and mitochondria. Furthermore, the mevalonate (MVA) pathway, using basic isoprene molecules, synthesizes terpenoid compounds in conjunction with the MEP pathway. The MEP pathway, integrated with the MVA pathway, produces terpenoid compounds in both the cytosol and the plastid cell compartments (Agostini-Costa et al., 2012; Twaij and Hasan, 2022). A comparison of SM biosynthesis across different crops is presented in **Table 2**. Plants have recently been viewed as bioreactors in terms of both targeted and scalable SM production as new technologies such as metabolic engineering and synthetic biology have emerged. Metabolic engineering focuses on multi-omics characterization, which includes a variety of genes and regulatory factors to better understand metabolic diversity. Synthetic biology expands on these understandings by developing *de novo* biosynthesis pathways and engineering efficient chassis organisms for metabolite production.

**Table 2:** Comparison of SM biosynthesis among different crops.

Pathway class: biosynthesis—secondary metabolite biosynthesis	*Beta vulgaris*	*Brassica napus*	*Brassica rapa*	*Hordeum vulgare*	*Saccharum* sp*ontaneum*	*Sorghum bicolor*	*Trifolium pratense*	*Zea mays*
α-Amyrin biosynthesis	✓	✓	✓				✓	✓
β-Carotene biosynthesis	✓	✓	✓	✓	✓	✓	✓	✓
β-Caryophyllene biosynthesis			✓	✓	✓	✓		✓
β-Cubebene biosynthesis						✓		
δ-Carotene biosynthesis	✓	✓	✓	✓	✓	✓	✓	✓
(+)-Camphor biosynthesis	✓				✓		✓	
(−)-Glycinol biosynthesis							✓	
(−)-Maackiain biosynthesis							✓	
(-)-Medicarpin biosynthesis							✓	
(3*E*)-4,8-Dimethyl-1.2.7-triene biosynthesis I	✓	✓	✓		✓	✓	✓	✓
(3*E*)-4,8-Dimethylnones-1,3,7-triene biosynthesis II		✓	✓					
(3*S*)-Linalool biosynthesis	✓	✓	✓	✓	✓	✓	✓	✓
(*E*,*E*)-4,8,12-Trimethyltrideca-1,3,7,11-tetraene biosynthesis	✓	✓	✓					
(*Z*)-Phenylmethanethiol *S*-oxide biosynthesis	✓							
2′-Deoxymugineic acid phytosiderophore biosynthesis				✓	✓	✓	✓	✓
2-Methylketone biosynthesis								✓
3,5-Dimethoxytoluene biosynthesis	✓						✓	
3-Methylthiopropanoate biosynthesis	✓	✓					✓	
8-*O*-Methylated benzoxazinoid glucoside biosynthesis								✓
9-Lipoxygenase and 9-allene oxide synthase pathway				✓				
Ent-kaurene biosynthesis I	✓		✓	✓	✓	✓	✓	✓
*trans*-Lycopene biosynthesis II (oxygenic phototrophs and green sulfur bacteria)	✓	✓	✓	✓	✓	✓	✓	✓
Abscisic acid biosynthesis	✓	✓	✓	✓	✓	✓	✓	✓
Abscisic acid biosynthesis shunt				✓				
Acetaldehyde biosynthesis I	✓	✓	✓	✓	✓	✓	✓	✓
Acetaldehyde biosynthesis II	✓		✓	✓	✓	✓	✓	✓
Anthocyanidin modification (Arabidopsis)	✓	✓	✓				✓	
Anthocyanin biosynthesis		✓		✓	✓	✓		✓
Apigeninidin 5-*O*-glucoside biosynthesis	✓	✓		✓	✓	✓	✓	✓
Avenacin biosynthesis, initial reactions	✓	✓	✓		✓	✓	✓	
B series fagopyrites biosynthesis from Fagopyrum esculentum (Buckwheat)	✓							
Barbaloin biosynthesis			✓		✓			
Baruol biosynthesis		✓						
Betacyanin biosynthesis	✓							
Betalamic acid biosynthesis	✓		✓					
Betaxanthin biosynthesis (via dopamine)	✓							
Betulinate biosynthesis	✓						✓	
Biochanin A conjugate interconversion							✓	
CA1P biosynthesis	✓	✓	✓	✓	✓	✓	✓	✓
Camalexin biosynthesis		✓	✓					
Chlorophyll cycle	✓	✓	✓	✓	✓	✓	✓	✓
Costunolide biosynthesis	✓			✓	✓	✓	✓	✓
Costunolide biosynthesis I	✓	✓			✓	✓	✓	
Crocetin esters biosynthesis					✓	✓		
Cyanidin 3,7-diglucoside polyacylation biosynthesis	✓			✓	✓	✓	✓	✓
Cycloartenol biosynthesis	✓	✓	✓	✓	✓	✓	✓	✓
Dhurrin biosynthesis		✓	✓		✓	✓	✓	✓
DIBOA-glucoside biosynthesis				✓	✓	✓	✓	✓
DIMBOA-glucoside biosynthesis	✓			✓	✓	✓		✓
diterpene phytoalexins precursors biosynthesis				✓		✓		✓
Epoxysqualene biosynthesis	✓		✓	✓	✓	✓	✓	✓
Eumelanin biosynthesis	✓	✓	✓	✓	✓	✓	✓	✓
Farnesene biosynthesis	✓	✓	✓					
Felinine and 3-methyl-3-sulfanylbutan-1-ol-biosynthesis	✓	✓	✓		✓	✓	✓	
Formononetin biosynthesis							✓	
Furaneol and mesifurane biosynthesis					✓		✓	
GA 12 biosynthesis	✓	✓	✓	✓	✓	✓	✓	✓
Geraniol and geranial biosynthesis	✓			✓	✓	✓	✓	✓
Geranyl acetate biosynthesis							✓	
Germacrene biosynthesis	✓	✓		✓	✓		✓	✓
Gibberellin biosynthesis I (non c-3, non, c-13 hydroxylation)	✓	✓	✓	✓	✓	✓	✓	✓
Gibberellin biosynthesis II (early C-3 hydroxylation)		✓	✓				✓	✓
Gibberellin biosynthesis III (early C-3 hydroxylation)	✓	✓	✓	✓	✓	✓	✓	✓
Glucosinolate biosynthesis from dihomomethionine		✓	✓					
Glucosinolate biosynthesis from hexahomomethionine		✓	✓					
Glucosinolate biosynthesis from homomethionine		✓	✓					
Glucosinolate biosynthesis from pentahomomethionine		✓	✓					
Glucosinolate biosynthesis from phenylalanine		✓	✓					
Glucosinolate biosynthesis from tetrahomomethionine		✓	✓					
Glucosinolate biosynthesis from trihomomethionine		✓	✓					
Glucosinolate biosynthesis from tryptophan		✓	✓					
Glycyrrhetinate biosynthesis				✓			✓	
Gramine biosynthesis				✓				
Hordatine biosynthesis								
Hydroxycinnamic acid tyramine amides biosynthesis								✓
Hydroxylate mugineic acid phytosiderophore biosynthesis				✓	✓	✓		✓
Indican biosynthesis					✓	✓		✓
Indigo biosynthesis					✓	✓		✓
Isoflavonoid biosynthesis I							✓	
Isoflavonoid biosynthesis II							✓	
Jasmonic acid biosynthesis	✓	✓	✓	✓	✓	✓	✓	✓
l-Nicotianamine biosynthesis	✓	✓	✓	✓	✓	✓	✓	✓
l-Tryptophan degradation Vl (via tryptamine)		✓		✓	✓	✓		✓
Laudanine biosynthesis	✓							
Leucine-derived hydroxynitrile glucoside biosynthesis				✓				
Linamarin biosynthesis							✓	
Lipid-dependent phytate biosynthesis I (via Ins(1,4,5) P3)	✓					✓	✓	✓
Lipid-dependent phytate biosynthesis II (via Ins(1,3,4)P3)		✓	✓					
Lupeol biosynthesis	✓	✓	✓	✓	✓		✓	
Lutein biosynthesis	✓	✓	✓	✓		✓	✓	✓
Luteolinidin 5-*O*-glucoside biosynthesis	✓	✓		✓	✓	✓	✓	✓
Methylerythritol phosphate pathway I	✓		✓	✓	✓	✓	✓	✓
Methylerythritol phosphate pathway II	✓	✓	✓	✓	✓	✓	✓	✓
Mevalonate pathway I	✓	✓	✓	✓	✓	✓	✓	✓
Momilactone A biosynthesis		✓			✓	✓		✓
Neoxanthin biosynthesis	✓		✓	✓	✓	✓	✓	✓
Olivetol biosynthesis	✓	✓	✓		✓	✓	✓	
Oryzalexin A, B, and C biosynthesis		✓						
Oryzalexin D and E biosynthesis								
Papaverine biosynthesis								
Pelargonidin conjugates biosynthesis		✓						
Phenylethyl acetate biosynthesis				✓				✓
Phytocassanes biosynthesis, shared reactions				✓	✓			
Phytol salvage pathway	✓	✓	✓	✓		✓	✓	✓
Phytosterol biosynthesis (plants)	✓	✓	✓	✓	✓	✓	✓	✓
Phytyl diphosphate biosynthesis	✓	✓	✓	✓	✓	✓	✓	✓
Resveratrol biosynthesis	✓	✓	✓	✓	✓	✓	✓	✓
Rubber biosynthesis							✓	
Sakuranetin biosynthesis						✓		✓
Salidroside biosynthesis			✓	✓	✓		✓	
Salvianin biosynthesis		✓		✓	✓	✓	✓	✓
Sanguinarine and macarpine biosynthesis		✓						
Shisonin biosynthesis				✓	✓	✓	✓	✓
Soybean saponin I biosynthesis	✓	✓					✓	
Spermidine hydroxycinnamic acid conjugates	✓	✓	✓	✓		✓	✓	✓
Steviol biosynthesis								✓
Steviol glucoside biosynthesis (rebaudioside A biosynthesis)	✓				✓		✓	✓
Ternatin C3 biosynthesis	✓				✓		✓	✓
Ternatin C5 biosynthesis		✓		✓	✓			
Thalianol and derivatives biosynthesis		✓	✓					
Traumatin and (*Z*)-3-hexen-1-yl acetate biosynthesis	✓	✓	✓	✓	✓	✓	✓	✓
Ursolate biosynthesis	✓						✓	
Usnate biosynthesis	✓	✓	✓		✓	✓	✓	
Valencene and 7-epi-α-selinene biosynthesis	✓	✓	✓		✓	✓	✓	
Violaxanthin, antheraxanthin, and zeaxanthin interconversion	✓	✓	✓	✓	✓	✓	✓	✓
Violdelphin biosynthesis	✓			✓	✓	✓	✓	✓
Zealexin biosynthesis								✓
Zeaxanthin biosynthesis	✓	✓	✓	✓	✓	✓	✓	✓

SM, secondary metabolite.Shading represents presence of a particular metabolite in the Plant species indicated.

### 3.2 Secondary metabolites in plant defense (physiological, biochemical, and molecular perspective)

**Figure 2:**
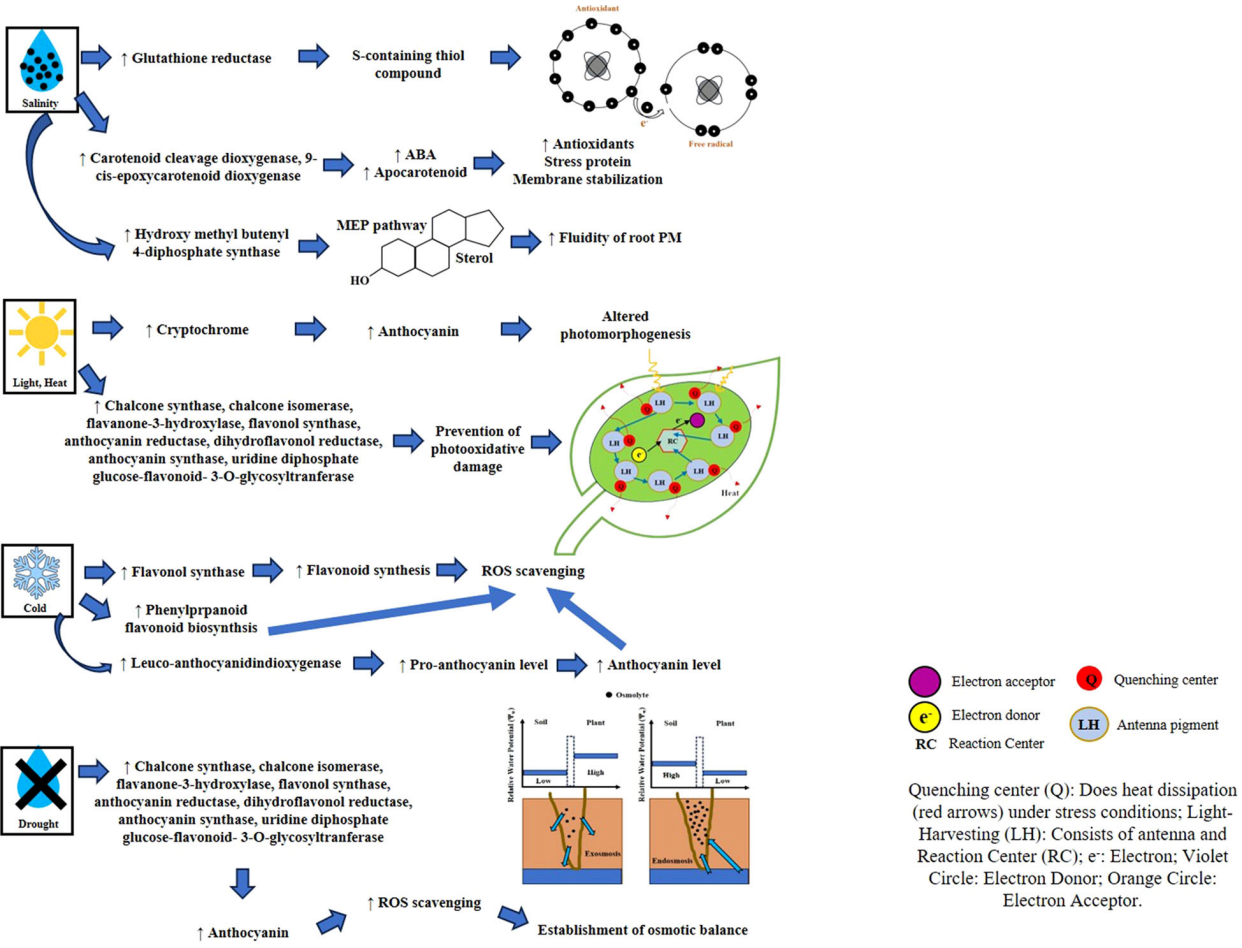
Roles and responses of SMs under different abiotic stresses. SMs, secondary metabolites.

**Figure 2** illustrates the roles and responses of various SMs under different abiotic stresses.

#### 3.2.1 Temperature stress

Heat stress (HS) has predominantly affected crop production and food security. Fodder crops, crucial for ensuring food security, supporting livestock, and maintaining environmental stability, face significant challenges due to high temperatures, posing a substantial threat to the livestock industry (Indu et al., 2023). Alfalfa (*M. sativa* L.), an exclusively cultivated leguminous forage crop, faces HS as a substantial limiting factor for its growth and development (Wassie et al., 2020). Arshad et al. (2020) conducted a study where alfalfa was subjected to HS conditions (40°C) and investigated changes in phenolic compound accumulation. The findings revealed an increase in the accumulation of flavonoids and phenolic compounds under HS as compared to the control plants. Moreover, flavonoids contribute to the regulation of antioxidant enzyme activities, enhancing the overall antioxidant defense system. Furthermore, miR156 has an important role in alfalfa thermotolerance, as it is involved in carbon, polysaccharides, and secondary metabolism and belongs to MYB, ABA response element-binding factor, WRKY, and heat shock transcription factor (Arshad and Hannoufa, 2022). Another important fodder crop, maize, is found to be severely affected by HS, and it reported that the Sultan (heat-tolerant) maize variety exhibits superior shoot growth and water content under HS alongside total soluble phenolics, flavonoids, tannins, and saponins that provide thermotolerance (Arshad and Hannoufa, 2022).

Phenolic compounds, especially flavonoids, play a crucial role in ameliorating HS in *P. purpureum* Schum. Jin et al. (2023) reported the evolution and expansion of the RWP-RK gene family, having a direct role in synthesizing phenylpropanoid SMs. These metabolites are reported to be directly involved in removing the harmful free radicals in plants and improving plant tolerance under heat, drought, and salt stress. Research studies have clearly indicated that the RWP-RK gene family improves heat tolerance in fodder crops by enhancing the antioxidant levels (peroxidase) and nitrogen use efficiency. The promoter region of the gene family (RWP-RK) consists of a significant number of methyl jasmonic acid (MeJA) and ABA-responsive elements contributing to the response mechanism under HS. The findings establish a scientific foundation for investigating the heat adaptation mechanism in fodder crops (Napier) and improving the heat tolerance mechanism in other fodder crops. A study conducted in bentgrass using metabolic profiling noted that heat-tolerant *Agrostis scabra* maintains higher accumulation or lesser degradation of soluble monosaccharides, branched-chain amino acid, oxoproline, and putrescine, having a role in energy production, secondary signaling, antioxidant defense, and osmotic regulations (Xu et al., 2013). Likely, the metabolic and transcript profiling in *Panicum maximum* Jacq. under elevated CO_2_ and temperature revealed a higher accumulation of sinapic acid, phenylalanine, and α-tocopherol, providing tolerance against stresses (Wedow et al., 2019). The NOL (non-yellow COLORING-1 like gene) gene accelerated heat-induced leaf senescence, and the knockdown of this gene in perennial ryegrass (*L. perenne*) produced the stay-green phenotype (Lei et al., 2021). Further, they noted that metabolic reprogramming of respiration, secondary metabolism, antioxidant defense, and protein synthesis pathways is involved in the regulation of heat tolerance. Therefore, it was established that SMs are crucial to mitigate HS in forage crops, as they regulate antioxidant defense, signaling pathways, and transcription factors. Heat stress tolerance is shown in various forage and non-forage crop studies that have focused on the role of differentially expressed metabolites, several of which have been identified as belonging to the SM class using Kyoto Encyclopedia of Genes and Genomes (KEGG) analysis. The vast majority of such metabolites were classified either as SMs (betaine, biotin, ABA, SA, or *trans*-zeatin) or as lipid-like compounds. Temperature-specific KEGG pathway analysis revealed overexpression of CoA biosynthesis and phenylpropanoid biosynthesis, which produce salicylic and glutamic acid (antioxidant defense), abscisic and JA (heat-stress signaling), betaine, and proline (osmoprotection) (Tian et al., 2020; Su et al., 2021; Zhou et al., 2024). Studies on major crop systems such as rice have also revealed the role of flavonoids (kaempferol and quercetin) in ROS detoxification and cellular structure stabilization, preventing damage to photosynthetic pigments under heat stress and ensuring a steady state in photosynthetic efficiency (Jan et al., 2021).

#### 3.2.2 Heavy metal stress

Heavy metals, primarily environmental pollutants, originate from anthropogenic sources such as mining as well as industrial sewage and sludge, herbicide, fungicide, insecticide, and animal manure. The soil is the primary nutrient source for plants, and the accumulation of HMs reduces the land quality and contaminates land, water, and crops (Singhal et al., 2023b). However, perennial grasses have a high tolerance potential against HM stress and are used for phytoremediation (Rabêlo et al., 2018). However, higher concentrations of HMs negatively influence the crucial functional processes and forage quality, which may further impact livestock health. Rezaeian et al. (2020) conducted a study on cadmium (Cd) pollution from industrial activities. They investigated the accumulation of Cd in alfalfa plants and noticed toxicity symptoms in cows’ livers and kidneys (Singh et al., 2024). In alfalfa, the application of increasing concentrations of Pb progressively reduced the root length and noticed an increased activity of the PAL enzyme (Ghelich et al., 2014). Further, the medicarpin (derivative of isoflavonoids) biosynthesis was increased in the shoot but decreased in root tissues, which also differentially altered the expression of CHS and vestitone reductase (VR) transcripts. A recent study was conducted in fountain grass (*Pennisetum alopecuroides*) and found that various metabolic pathways coordinate to regulate Cd tolerance. Further, it was noticed that genistein, *p*-coumaric acid, and 3-hydroxy-4-methoxycinnamic acid were differentially shared in the root and leaf tissues and mostly related to oxidative stress mitigation (Mi et al., 2023). Likely, the sensitive genotype (WB-144) of Bermuda grass (*Cynodon dactylon* L.) showed an increment in the isoleucine and valine amino acid contents under Cd stress and subjected to stress tolerance through detoxification process or biosynthesis of chelating peptides (Xie et al., 2014). Also, in alfalfa, long-term exposure to Cd (5 months) imbalances the redox status of the cell and increases the abundance of iso(flavonoids) that detoxified the HM stress by increasing antioxidative defense and metal chelation (Gutsch et al., 2020). A study was conducted in *Stylosanthes guianensis* under Mn toxicity (400 µM) and found that the levels of total phenols, flavonoids, tannins, and anthocyanidins were enhanced by 1.1-, 1.6-, 2.1-, and 7.4-fold as compared to control (Jia et al., 2020). Moreover, they reported that the expression of PAL was upregulated under Mn toxicity, and genes (*CCR*, *CAD*, *COMT*s, F6Hs, and *POD*) encoding different steps in phenylpropanoid pathways were also upregulated.

However, the different SMs responded differentially to HM stress. For instance, in maize, total phenolic acid and chlorogenic acid showed a positive correlation with Pb. In contrast, total phenolic acid and ferulic acid were negatively expressed under Cu and Cd stress (Kisa et al., 2016). Likely, a root metabolic profiling was conducted in maize using two Huidan No. 4 (Pb tolerance) and Ludan No. 4 (Pb-sensitive) under Pb stress and found that phenylalanine was changed only in tolerant variety and that the increment was the highest (437-fold) as compared to other metabolites (Zhang H. et al., 2021). They also hypothesized that Pb tolerance in tolerant variety was linked to the sequestration of Pb in the cell wall, and phenylalanine was related to cell wall synthesis. The metabolomic profiling of sorghum crops under Cd stress revealed that 12 categories of metabolites were differentially expressed. Among them, flavonoids (20.84%), phenolic acids (17.04%), amino acids and their derivatives (12.6%), and alkaloids (10.7%) were highly abundant under Cd stress (Jiao et al., 2023). Further, investigations of genes and metabolites have revealed that the key genes involved in flavonoid biosyntheses such as naringenin 7-*O*-methyltransferase (NOMT), flavonoid 3′5-hydroxylase (F3′5′H), CHS, chalcone-flavanone isomerase (CHI), and flavonoid 3′-monooxygenase (F3M) were upregulated in Cd treatment. In *Panicum*, the application of high Cd concentration (1.5 mmol L^−1^) drastically reduced the photosynthesis rate (76%) by altering the stomatal conductance and causing a loss of quantum efficiency. However, the application of potassium (1.5 mmol L^−1^) upregulated polyamine synthesis, improving plant performance by strengthening antioxidant defense, regulating cell homeostasis and metal chelators, and acting as a stress signaling pathway component (de Anicésio and Monteiro, 2022). Therefore, it can be concluded that SMs have a key role in the mitigation of HM stress by minimizing ROS toxicity, maintaining redox homeostasis, and improving stress signaling pathways.

#### 3.2.3 Drought stress

Optimizing the concentration of SMs is one of the key mechanisms in safeguarding the cellular and sub-cellular structures and functions and developing resistance during drought stress. Various physicochemical mechanisms have been reported to play a crucial role in SM-based defense mechanisms during water deficiency in the forages. Physiological mechanisms such as cell osmotic adjustment, water use efficiency (WUE), and relative water content (RWC) by involving different groups of SMs in the crops like berseem (Iannucci et al., 2000; Balazadeh et al., 2021), multi-cut pearl millet (Rostamza et al., 2011), sorghum (Zheng et al., 2023), and oat (Xie et al., 2021; Wang et al., 2023; Xu et al., 2024) have been reported to provide tolerance during the low water stress. Some drought-induced volatile SMs alert affected plant tissues to acclimatize drought stress via systemic induction of drought signaling in forages. SMs like phenolic and glucosinolate derivatives are found to be involved in maintaining osmotic potential in roots as well as water uptake and transportation under water scarcity (Nicolas-Espinosa et al., 2023). Fariaszewska et al. (2020) studied the physiological and biochemical responses of nine forage grass varieties belonging to five species of *Festuca*, *Lolium*, and *Festulolium*. They reported the induced drought acclimatization via enhanced SMs like proline, phenols, flavonoids, and water-soluble carbohydrates under drought stress conditions.

Prolonged water scarcity induces oxidative stress in forage crops that trigger the generation of ROS. SMs, particularly flavonoids and polyphenols, are involved in ROS scavenging (Treml and Smejkal, 2016). Likewise, SMs like polyphenols, particularly phenolic acids, flavonoids, and tannins through regulative as well as anti-oxidative activities, are believed to have higher ROS scavenging during drought stress. In sorghum and oats, polyphenol-dependent ROS scavenging reduction of membrane lipid peroxidation through enhanced antioxidant activities upon stress has been reported by Getnet et al. (2015); Devnarain et al. (2016); Wu et al. (2016), and Ndlovu et al. (2021). In grass species *Cenchrus ciliaris* L. and *Cyperus arenarius* Retz., prolonged drought stress inhibited enzymatic functions, destroyed photosynthetic efficiency through ROS production, and also enhanced the ROS scavenging through SOD, peroxidase (POD), and CAT antioxidant activities that majorly involve SM compounds (Ghafar et al., 2021). A tolerant genotype of alfalfa exhibited more drought tolerance through over-accumulation of SMs like tryptophan, homocarnosine, *S*-adenosylhomocysteine, cytidine, and clairol, as compared to the sensitive one (Ma et al., 2021). Some of the primary metabolites like sugars and sugar alcohols playing secondary roles as compatible solutes or osmolytes exhibited over-accumulation during drought, which have been found associated with improved drought tolerance through osmotic adjustment and continued biomass production during water stress in sorghum (Ogbaga et al., 2016; Fariaszewska et al., 2020). Likewise, amino acids such as proline, methionine, lysine, and arginine were also found to have over-accumulated in the drought-tolerant perennial grasses like *Agropyron cristatum*, *Agropyron intermedium*, *Festuca ovina*, *Festuca arundinacea*, *C. dactylon*, *Bromus inermis*, and *Bromus confinis* (Khoshkholghsima and Rohollahi, 2015). Similarly, the altered profiling of plant lipids, particularly the glycerolipids and extracellular lipids, also acted as important signaling mediators in oats (Sanchez-Martin et al., 2018) and sorghum (Zhang X. et al., 2021) during prolonged drought stress. Further, the damaged plant systems initiate the natural repair mechanisms under drought by inducing molecules like LEA proteins and metabolites to mitigate the ill effects of the stress. Further, the reduced moisture availability has been linked to the formation of metabolite, i.e., dhurrin, an N_2_-containing glucoside, in seed grain and leaves of forage sorghum at an immature stage, making them cyanogenic for feeding livestock (Busk and Møller, 2002; Nielsen et al., 2016).

Molecular response to drought tolerance is a multi-genic trait governed by several genes. The altered gene expression and associated TFs and protein kinases involved in stress perception, signal transduction, and transcriptional regulatory networks lead to the accumulation of drought-responsive SMs in the tolerant forage genotypes under drought. Recently, the underlying molecular mechanisms and up- and downregulated Differentially Expressed Genes (DEGs), genes/gene families, and TFs were identified for osmotic stress using single-molecule real-time sequencing and Next Generation Sequencing (NGS) technique in forage crops like *Sorghum sudanense* (Piper) Stapf (Liu Q. et al., 2023), oat (Xu et al., 2024), and *Lolium multiflorum* (Liu Q. et al., 2022). The expression studies identified that the genes encoding TFs, namely, MAT, MYB, ERF, CBL, CCR, and NAC, may have a very crucial role in SM-mediated drought response in crop plants, including forages (Yadav et al., 2021). Unveiling the molecular mechanisms underlying drought tolerance in forages is nascent and requires further systematic investigations. Effective utilization of available high-throughput omics/NGS tools is highly desirable in identifying major underlying well-resolved quantitative trait loci (QTLs)/candidate genes and mechanisms for fluctuation in SM amount/concentration, which could significantly aid in future breeding forage crops for drought tolerance.

#### 3.2.4 Salinity stress

Saline stress, mainly associated with NaCl, Na_2_SO_4_, and other neutral salts, influences the plants by disturbing the cellular osmotic and ionic homeostasis, in response to which the plants produce different types of SM compounds in order to normalize the adverse effects through antioxidants, ROS scavengers, and regulatory molecules. High ion concentration (300 mM NaCl in oats) disrupted the cell membrane integrity, membrane lipid profiles, and membrane permeability in the sensitive forage cultivars, which disturbed the cytosolic osmotic adjustment and compartmentalization of ions during salinity stress (Zhang et al., 2022; Liu J. et al., 2023). In oats, the salinity tolerance was associated with high proline, soluble sugars, membrane stability, SM accumulation (phenol content), and antioxidant mechanisms involving POD, CAT, SOD, ascorbate peroxidase (APX), etc (Kumar et al., 2021; Chen C. et al., 2022; Zhang et al., 2022; Wang et al., 2022). In fodder maize, genes like *ZmSRG7*, *ZmBZ1*, *ZmNUP58*, and *ZmWRKY17*, controlling antioxidant activities during salinity stress, have been identified (Cai et al., 2017; Wei et al., 2022; Wang et al., 2022; Liu Z. et al., 2022). Similarly, alfalfa (*M. sativa*) exhibited over-accumulated saponins in the shoots. In contrast, *Medicago arborea* overexpressed saponins in the roots, whereas *Medicago alborea* exhibited an enhanced accumulation of lignans and phenyl tetrahydrofurans predominantly in the roots (Sarri et al., 2021).

The molecular mechanisms for salinity stress tolerance in forage crops primarily involved gene expression controlling ionic homeostasis right from poor uptake of Na^+^, higher exclusion of Na^+^ in the root system to its compartmentalization into root vacuoles, and absorption of more K^+^ ions. The altered gene expression initiated the biosynthesis of enzymes and molecules required for the movement of these ions in forage crops. Overexpression of SOD synthesis genes, i.e., *MnSOD* and *CuZnSOD*, under salt and alkali stress resulted in higher SOD antioxidant activities, which aided in ROS scavenging in oats (Bai et al., 2023). Fewer studies have clearly identified the responsive gene sequences/TFs involved in the mechanisms of different forage crops for mitigating salinity stress. For instance, the tolerant plants of oats exhibited the higher potential of avoiding root Na^+^ uptake, excluding more Na^+^, compartmentalization of excessive Na ^+^ into vacuoles, and assimilating more K^+^ through expressing *AsAKT1* and *AsHKT2*, *AsSOS1*, and *AsNHX1*, and *AsVATP-P1*, *AsKUP1*, and respectively, as compared to the susceptible ones (Zhang et al., 2022; Chen C. et al., 2022). Differential expressions of various families of TFs, including ZFPs, MYB, WRKY, NAC domain proteins, *bzZIP* TFs, and AP2 domain-containing TFs, were also reported under salinity stress in oats (Wu et al., 2018). Transcriptomic studies in sorghum identified *LOC8071970*, *LOC8067721*, *LOC110430887*, *LOC8070256*, and *LOC8056880* as potential candidate genes associated with SM-based salt stress tolerance (Jeon et al., 2023). Multi-omics analysis recognized DEGs associated with the biosynthesis of flavonoids, phenylpropanoids, arginine, and proline metabolism that play a very crucial role in sorghum to withstand salinity stress (Ren et al., 2022). Further, the altered hormonal signaling involving ABA, JA, and SA pathways under salinity stress yielded stress-responsive DEGs in the sorghum genotypes (Chen G. et al., 2022). Although forage includes several traditional and non-traditional grasses, only major crops like oats, sorghum, maize, and pearl millet, have been predominantly explored for molecular studies, including genome-wide association studies (GWASs) and multi-omics. These approaches have resulted in the identification of stress-responsive gene families and TFs with high potential utility for futuristic forage breeding for salinity tolerance. Nevertheless, the novel NGS tools could contribute enormously to comprehensive insight into these complex salinity tolerance mechanisms in different species of forage grasses.

#### 3.2.5 Combined and multiple stress

As climate change intensifies, plants in the field are increasingly subjected to multiple and combined stresses, which often exert a more severe impact on their growth and survival compared to individual stresses. A recent review focused on the defensive roles of SMs in maize crops. Further, they discussed that SMs are involved in the regulation of biochemical pathways and TFs and participate in abiotic and biotic stress tolerance (Chávez-Arias et al., 2022). Likely, Goyal et al. (2023) reported that SMs participated in abiotic stress defense pathways by regulating metabolites, oxidative stress, cell homeostasis, and stress-responsive elements in forage crops. An experiment was conducted in *P. maximum* Jacq. (Guinea grass) under elevated CO_2_ (600 μmol mol^−1^) and temperature (+2°C) and found alterations in transcript and metabolic profile related to SMs and stomatal functions (Wedow et al., 2019). Changes in CO_2_ and temperature influence plant SM synthesis by influencing physiological and metabolic responses to stress. Elevated CO_2_ levels often improve photosynthesis and carbon availability, perhaps enhancing the synthesis of SMs, particularly those engaged in defense. However, in other situations, plants may choose growth above protection, resulting in lower levels of particular metabolites.

A study was conducted in Creeping Bentgrass using acibenzolar-*S*-methyl (ASM) under combined drought and heat stress. It was found that ASM improves stress by enhancing protein synthesis and metabolite accumulation involved in osmotic adjustment, energy metabolism, and stress signaling (Bai et al., 2023). Therefore, SMs are crucial for singular stress and have important roles in multiple abiotic stress tolerance, and the mechanism of stress tolerance through SMs is highlighted in **Table 1**.

## 4 Biotechnological strategies for secondary metabolite production to improve forage crop stress tolerance

### 4.1 Omics approaches

The advent of omics approaches and their integration provided a multi-omics platform for climate-resilient crop generation. Forage crops have been neglected in this aspect, with very few studies on crop improvement (Kumar and Bhat, 2014) and complex genomes of some crops like alfalfa. Multi-omics includes genomics, transcriptomics, proteomics, metabolomics, and phenomics.

Genomics includes genome sequencing and helps study environment-associated multigenic traits (Pérez-de-Castro et al., 2012). Different forage crops were sequenced, including sweet sorghum (Cooper et al., 2019), cowpea (Lonardi et al., 2019), maize (Schnable et al., 2009), oats (Kamal et al., 2022), and alfalfa (Shen et al., 2020) with the creation of extensive sequencing data generating databases and genomic resources for crop improvement. These sequencing data provide the resource for genomic variation, QTL identification, and GWAS. Complex traits require advanced genomic methods like GWAS and association mapping, which provides whole-genome coverage and better marker–trait variation explanation than linkage mapping (Chakradhar et al., 2017). Minicore collection of different genotypes makes accessing the variations for population improvement easy. For example, the University of California-Riverside Minicore (UCR Minicore) is a minicore collection of cowpeas (Muñoz-Amatriaín et al., 2021). For instance, quality traits single-nucleotide polymorphisms (SNPs) were identified in sorghum by genotyping 245 accessions under different environmental conditions (Li J. et al., 2018). Genotyping by sequencing (GBS) is another genomics tool for forage improvement, as shown in maize with 196 SNPs associated with forage quality traits (Zhao et al., 2018). Biomass accumulation is an essential trait in forage crops, which is essentially contributed by cell wall biosynthesis compounds such as cellulose, hemicellulose, and pectin. In sorghum, genomics studies have identified 20 different gene families with 520 gene members linked with cell wall biosynthesis (Rai et al., 2016). Alfalfa breeding populations were mapped by association mapping for stem digestibility and biomass yield using simple sequence repeat (SSR) markers (Li et al., 2011).

Forage crops benefit from enhanced sugar accumulation, which is crucial for improving their quality as fodder. Key traits associated with increased sugar content include brix value, stem juiciness, sugar yield, and juice volume. In various grain sorghum cultivars, a combination of GWAS and QTL mapping has identified genetic variations linked to these traits (Li Y. et al., 2018). The genetic variation also plays a role in lignin accumulation traits. For example, the brown midrib (bmr) trait, associated with four alleles (*bmr2*, *bmr6*, *bmr12*, and *bmr19*), has been identified in sorghum. These alleles contribute to increased biomass (*bmr2*, *bmr6*, and *bmr19*) and lignin accumulation (*bmr12*) (Da Silva et al., 2018). Similarly, in maize, bm3 mutants exhibit improved fodder quality, which is controlled by the caffeic acid *O*-methyltransferase (COMT) locus (Barrière and Argillier, 1993). Other important forage traits, such as leaf length, dry weight, leaf number, leaf width, and overall green fodder yield, can be studied through genome-wide analysis or sequencing, as these traits have a significant impact on fodder productivity. Notably, in sorghum, the stay-green trait, controlled by four QTLs (Stg1, Stg2, Stg3, and Stg4), has been identified as key to understanding the molecular mechanisms underlying drought resistance and sustained green foliage (Harris et al., 2007).

Fodder quality was improved in maize by manipulating BMR, wx, Leafy1 (*Lfy1*), and floury-2 genes, and silage hybrids were developed (Pinter et al., 2012). Similarly, different QTLs for leaf shape, pod length variation, flowering time, seed-related traits, and disease resistance were identified in cowpea. For example, three QTLs for bacterial blight resistance in cowpea qtlblb-1, qtlblb-2, and qtlblb-3, were identified, which could be used in breeding programs (Dinesh et al., 2016). GWAS was utilized for oat crown rust to identify race-specific resistance genes (*Pc38*, *Pc39*, and *Pc48*) (Wight et al., 2005). Further, Mrg01, Mrg03, Mrg08, Mrg20, Mrg23, and Mrg28 linkage groups have different QTLs associated with crown rust resistance (Winkler et al., 2016). Marker-based studies have also elucidated the diversity of forage germplasms and helped to establish linkage maps (Boukar et al., 2016). For example, in cowpea, different advanced marker systems like SNPs and SSRs were used for genetic variation, origin, and domestication (Ghalmi et al., 2010). Similarly, 442 cowpea landraces were genotyped by 1,500 SNP markers, revealing two major gene pools of cultivated African cowpea landraces (Huynh et al., 2013). Different linkage maps derived from the molecular markers presented the opportunity for better resolution, map-based cloning, association mapping, genotyping by sequencing, and GWAS in forage crops. For example, GWASs in cowpea significantly identified the variations in pod length and root architecture (Xu et al., 2017; Burridge et al., 2017). Comparative genomics has also been utilized on cowpea, soybean, and alfalfa crops for microsynteny analysis, revealing its similar genomic region governing the fodder quality and yield (Kulkarni et al., 2018). Synteny studies between oats, rice, and *Brachypodium distachyon* reveal linkage groups, providing information about different desired traits in oats (Gutierrez-Gonzalez and Garvin, 2011).

Transcriptomics utilizes RNA sequencing and transcript profiling to determine the gene expression in forage crops. It is often used to understand gene expression profiling under various environmental conditions, nutrient profiling, morphological traits, and stress conditions (Cramer et al., 2011; Varoquaux et al., 2019). For example, a sweet sorghum transcriptomic study reveals that under salinity and water lodging stress, better sugar accumulation increased growth rate and biomass accumulation (Ngara et al., 2021). These sugar accumulation phenotypes are contributed by sugar transporter genes (*SbSUT1*, *SbSUT2*, and *SbSUT6*) expression in sweet sorghum genotypes (Kanbar et al., 2021). Similarly, stress, growth, and development-related genes were characterized by cowpea transcriptomic approaches, revealing their role in pod and seed development (Yao et al., 2016).

Proteomics analysis provides information about different proteins, their concentrations, and changes in them due to post-translational modification with their functional localization by mass spectrometry, 2D gel electrophoresis, and matrix-assisted laser desorption ionization–time-of-flight–mass spectrometry (MALDI–TOF–MS) (Mustafa and Komatsu, 2021). For example, key proteins were identified in sorghum for their post-flowering drought stress tolerance and examined using proteomics tools (Woldesemayat et al., 2018). In cowpea, differential proteome response under salt stress was observed, with 22 proteins involved in stress recovery, maintaining energy metabolism, and photosynthesis (de Abreu et al., 2014). Maize proteomic studies reveal changes during Asian corn borer infection with the activation of defense-responsive proteins like pathogenesis-protein 1 (Zhang et al., 2015). In oats, increased ascorbate and glutathione levels were detected in response to powdery mildew resistance (Zechmann, 2020). Comparative proteomics analysis in alfalfa plants reveals an accumulation of differential proteins in drought-sensitive and tolerant varieties, which will help to understand their role in adaptive mechanisms adopted by the plant (Zhang and Shi, 2018).

Metabolomics involves identifying functional metabolites essential in modulating the biochemical processes, resulting in phenotypic expression under different conditions (Führs et al., 2009). For example, in sorghum under drought stress, changes in metabolites like lipids, amides, and total carbohydrates enable the plant to adjust its cellular metabolism under stress (Rajarajan et al., 2021). Earlier metabolomics was used to study plant environment interactions with breeding applications. Hydrogen cyanide (HCN) in fodder sorghum, produced from dhurrin, is an essential targeted trait. Fourteen genes related to Dhurrin metabolism were identified by combining transcriptomic and metabolomic studies in sorghum, which could be used as a criterion for selecting sorghum varieties (Choi et al., 2020). Similarly, in cowpea, polyphenols and carotenoids were identified through metabolomic approaches such as electrospray ionization–mass spectrometry (ESI–MS), gas chromatography–mass spectrometry (GC–MS), gas chromatography, and high-performance liquid chromatography (HPLC), which provided a significant approach for enhancing this pathway metabolite production (Yeo et al., 2018). Male sterility-related metabolite accumulation under heat stress in maize revealed male sterility-related mechanisms (Begcy et al., 2019). During salt stress in oats, differential metabolite accumulation determines the susceptibility and tolerance phenotype (Xu et al., 2021). Similarly, the metabolomic approach revealed nutrient changes in alfalfa plants (Fan et al., 2018). These approaches could be used according to forage crop traits to determine the underlying variations for crop improvement.

Phenomics includes using tools to measure phenotypic and physiological characteristics under different environmental conditions, as shown in the case of fodder crops (Arya et al., 2022). These tools include imaging sensors, thermal imaging, multispectral imaging techniques, manned aircraft, and aerial phenotyping platforms such as unmanned aerial vehicles (UAVs) to measure canopy temperature, nitrogen content, plant height, and chlorophyll content (Watanabe et al., 2017). Interestingly, mobile robots were reported to record the traits of sorghum (Young et al., 2019). Phenomics application has also been reported in maize for drought stress conditions (Wu et al., 2021), in oats for biotic and abiotic stress (Nogueira et al., 2007), and in alfalfa for biomass (Feng et al., 2020). Phenomics cost and error rate drastically reduce its large-scale application in different forage crops. The technological improvement with robust analytics tools and integration of omics platforms present eco-friendly, low-cost, and rapid methods for forage crop improvement.

Earlier transcriptomic studies on heat responses have shown that most heat-responsive genes are involved in basic metabolic processes, including photosynthesis, respiration, protein biosynthesis, and hormone signaling, and also heat stress response-related transcription factors, including heat shock factors (Mondal et al., 2024). Comparative transcriptomic studies under prolonged heat stress in genotypes with different degrees of heat tolerance, especially in stress-tolerant grass plants, may potentially identify some heat tolerance-related genes involved in genetic variation. This knowledge is of critical importance for the use of heat tolerance-regulating genes in genomic engineering or molecular breeding schemes. Proteomics has examined stress-responsive enzymes involved in the biosynthesis of SMs, including PAL and chalcone synthase, that enhance antioxidant defense. In parallel, metabolomic profiling has examined enhanced accumulation of osmolytes like proline and glycine betaine, and flavonoids and phenolic acids, which enhance stress tolerance by preventing oxidative damage and cellular turgor maintenance (Mekuria et al., 2025).

### 4.2 Genome editing

The precise manipulation of genomes through clustered regularly interspaced short palindromic repeats (CRISPR) and CRISPR-associated nuclease 9 (CRISPR-Cas9) revolutionized the plant genome editing application (Jinek et al., 2012). Different model crops have been edited through CRISPR-Cas9; however, there are minimal reports on forage crops (Jiang et al., 2013; Shan et al., 2013). In green foxtail millet (*Setaria viridis*), a homolog of the *indeterminate 1* (*ID1*) gene from maize was targeted in foxtail millet line 193-31, resulting in delayed flowering. Genome editing is mainly carried out by generating knockouts with gRNA designed by different systems such as CRISPR-Cas9 and CRISPR-Cas12a, for example, in *Lolium arundinaceum* and *S. viridis* (Zhang L. et al., 2021; Weiss et al., 2020). Regulatory variations of these enzymes, like dCas9, with specific DNA binding ability, can affect endogenous genes’ transcriptional levels, which can also create gain-of-function mutants (Ding et al., 2022).

Mechanistically, genome editing tools such as CRISPR/Cas9 enable specific gene editing of key biosynthetic enzymes of SMs. For example, PAL, the central enzyme of the phenylpropanoid pathway, can be manipulated to enhance phenolic compound production to boost antioxidant capacity upon oxidative stress. Likewise, editing of CHS can improve flavonoid biosynthesis to boost ROS detoxification and defend against photosynthetic apparatus upon heat or drought stress (Sustek-Sánchez et al., 2023). Moreover, TFs such as MYB, WRKY, and bHLH, involved in SM biosynthesis regulation, can be edited to optimize metabolic flux and improve metabolite accumulation under stress conditions. Genome editing can optimize the abiotic stress response of the plant by the activation of stress-inducible promoters or the inhibition of negative regulators and therefore improve stress tolerance with minimal impact on biomass and forage quality (Capdeville et al., 2023).

Candidate genes identified through omics approaches for abiotic stress, nutritional content, cell wall biosynthesis, etc., could be manipulated through the CRISPR technology in forage crops. For example, heat sensors like CNGCs become activated in the heat stress fluidity of membranes, which could be used for stress tolerance (Niu and Xiang, 2018). Similarly, heat shock proteins (HSPs) and heat shock factors (HSFs) in the forage crops will be candidates for heat stress tolerance (Yan et al., 2017). Other candidate genes that elevate the expression from its basal state to cope with stress include kinases, which regulate post-translational modification of other stress pathway genes, leading to their activation in stress response (Damaris and Yang, 2021). For example, overexpression of *A. cristatum* forage grass gene AcSnRK2.11 in tobacco leads to increased survival and recovery of plants under cold stress by activating dehydrin genes (Xiang et al., 2020). Downregulating the negative regulators of stress responses is one such approach in genome editing that improves stress tolerance. For example, knockout mutant wheat Tasa11 has smaller and fewer stomata to cope with drought conditions (Abdallah et al., 2022). Similarly, genome editing could alter transporter genes to provide salinity tolerance in forage grasses (Alam et al., 2022).

A strong gametophytic self-incompatibility (SI) system in forage crops is one of the most significant reproductive features, making inbreeding difficult. Therefore, genome editing could modify the multi-allelic s and z loci, governed by *DUF247* genes controlling the SI (Herridge et al., 2022). These genes could be knocked out to develop self-compatibility, which will help produce improved cultivars of these crops through hybrids, as shown in the case of wheat (Singh et al., 2018; Okada et al., 2019). Here, the male sterility 1 (*Ms1*) and *Ms45* genes were edited to produce male sterile lines in wheat. Generation of double haploids through MTL gene knockout is also possible in forage crops, which will help reduce the time taken in forage crop breeding, as shown in the case of foxtail millet (*Setaria italica*) (Cheng et al., 2021). Apomixis is another vital trait that helps fix hybrid vigor. Inducing mutations in meiotic control genes by genome editing, clonal diploid gametes were produced in rice, which later on, through parthenogenesis induction, produce the clonal progeny (Khanday et al., 2019; Zhu et al., 2020).

Identification of candidate genes involved in abiotic stress responses is vital for the progress of molecular breeding programs in forage crops. Recent studies by Mondal et al. (2024) emphasized that stress tolerance in forage species is controlled by numerous genes, several of which are tightly associated with SM biosynthesis pathways that play roles in adaptive stress mitigation mechanisms. A set of genes is listed in **Table 1**, which are reported to be expressed under stress conditions in some other studies. These genes can be utilized as candidate genes for further genetic manipulation, which can provide improved stress tolerance to fodder and forage crops. Genome editing has enormous opportunities for forage grass modification and their improvement with speed and multiplexing targeting abilities. CRISPR-Cas could also be used for complex trait modifications. However, the lack of *in vitro* transformation protocols, the availability of adequately annotated reference genome sequences, and gRNA design could be possible bottlenecks in forage crop modification. These bottlenecks should be considered before designing any genome editing program for forage crops. The integration of CRISPR-Cas technology with omics approaches and modern plant breeding forage crops could be significantly improved (Pandey et al., 2022).

### 4.3 Bioengineering of metabolic pathways to improve forage crops

**Figure 3:**
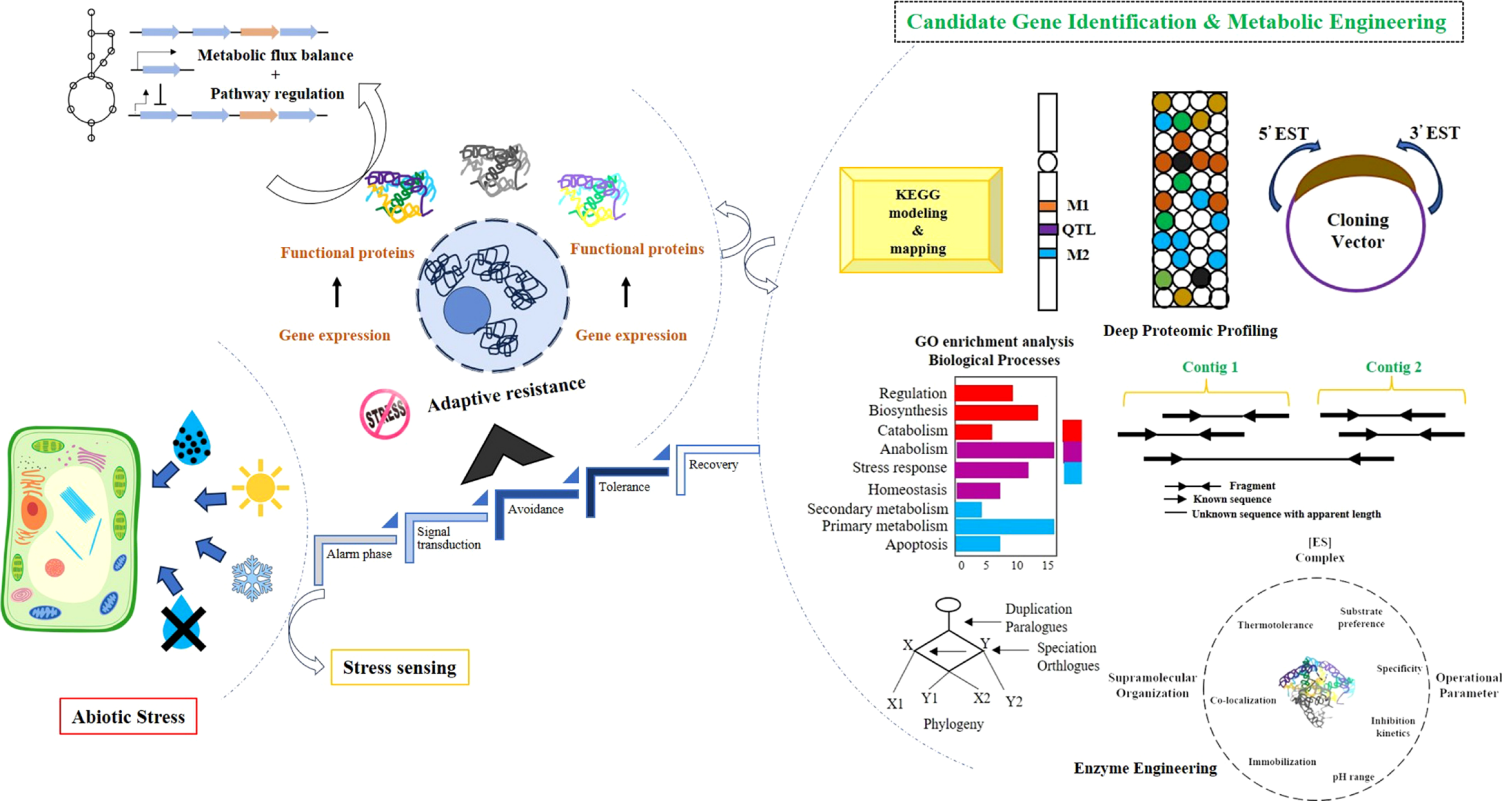
Enhancing stress sensing through candidate gene identification and bioengineering strategies.

**Figure 4:**
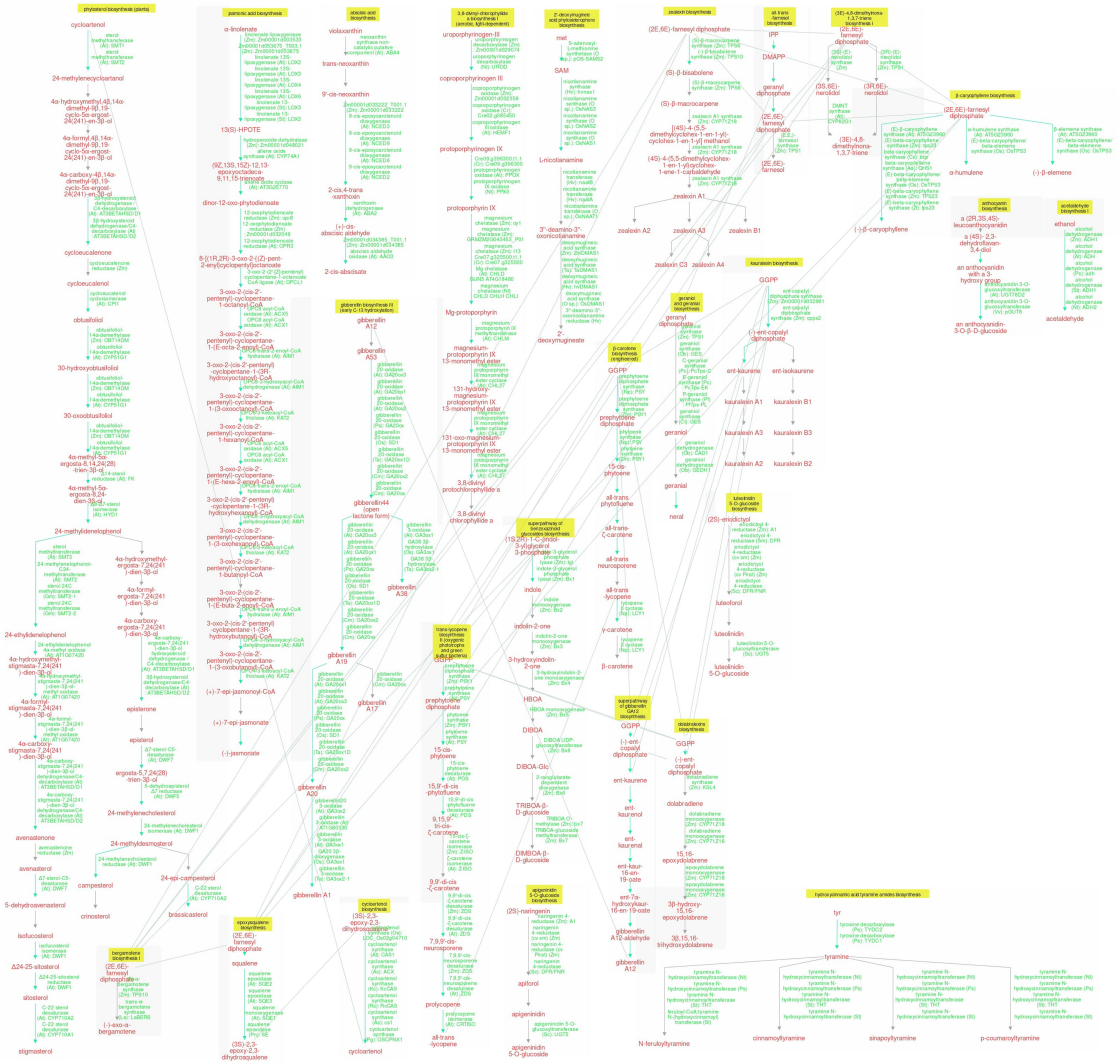
Graphical representation of abiotic stress signaling and role of biotechnological interventions in forage crop improvement through SM production. SM, secondary metabolite.

Forage crops are a rich source of organic matter and other nutritional components but require modification in their composition for better utilization as energy crops. They have different biochemical components in their cell wall and plant structures, like fructans, which could be used for better digestibility. For example, fructosyltransferase expression in ryegrass results in an increase in water-soluble carbohydrates, leading to better biomass accumulation and lower neutral detergent fiber with improved metabolizable energy in transgenic lines (Badenhorst et al., 2018). Similarly, lignin modification in the cell wall improves digestibility, an essential trait for improved forage crops (Grabber et al., 2019). For example, it has been shown that targeting lignin biosynthesis genes improves digestibility without changing the plants’ yield (Halpin, 2019). Transgenic maize with COMT antisense cassette significantly decreases lignin content at the flowering stage (Piquemal et al., 2002). In alfalfa, the downregulation of the cinnamate 4-hydroxylase (*C4H*) gene reduces the lignin content (Reddy et al., 2005). The first genetically engineered commercial forage crop, HarvXtra, was produced by knocking down the expression of caffeoyl-CoA 3-*O*-methyltransferase, leading to reduced lignin content and better digestibility (Barros et al., 2019). Additionally, reducing the resistant starch improves the digestibility of forage crops in livestock because resistant starch inhibits complete digestion (Raigond et al., 2015). These reports suggest ample opportunities for forage crop improvement through multi-omics, genome editing, and bioengineering pathway modifications. **Figure 3** illustrates the enhancement of stress sensing achieved through the identification of candidate genes and bioengineering approaches. Advancements in technologies combined with the identification of key candidate genes will facilitate cost-effective and feasible modifications in forage crops in the near future, as depicted in **Figure 4**.

### 4.4 Limitations

Most forage and fodder crops either are polyploid or contain complex genomes. Gene redundancy and allelic variation make it challenging to identify accurate targets and edit them, sometimes necessitating concurrent modification of several copies of a gene to see any noticeable phenotypic effect (Nadon and Jackson, 2020; Thriveni et al., 2024). Stress tolerance in forage and fodder crops is identified as a multigenic trait, which is crucial for increasing resistance to abiotic stresses like drought and salinity. Recent developments in molecular breeding and genetic engineering have been aimed at the identification and manipulation of several genes related to stress tolerance to enhance forage crop varieties. The subsequent sections discuss important points regarding this topic. Abiotic stress responses are mediated by intricate gene networks, epigenetic regulation, and cross-talk among pathways. Single-gene edits tend to yield restricted or context-dependent tolerance, and therefore, it is essential to adopt multiplexed editing or gene stacking strategies, which require much trial and error, considering unexplored genomes. The production of SMs is controlled by intricate, linked biosynthetic pathways with more than one gene, transcription factors, and environmental interactions. In forage crops, these pathways tend to be poorly described, and specific gene targets are hard to determine without unforeseen effects on plant fitness or productivity (Liras and Martín, 2023). The regulation of SM biosynthesis under stress tends to be polygenic and is controlled by both genetic and epigenetic components. Modification of individual genes is unlikely to result in substantial, stable improvements in metabolite accumulation, particularly when growing under field conditions where several stress factors cooperate (Jan et al., 2021; Li et al., 2024).

## 5 Conclusion

### 5.1 Key findings

The comprehensive exploration of the impact of abiotic stresses on forage crop growth and development, coupled with the intricate role of SMs in stress mitigation, underscores the urgency of devising strategies to enhance crop resilience and sustainability. Abiotic stresses such as drought, salinity, extreme temperatures, and heavy metal toxicity significantly impede plant vigor and productivity, compromising growth, yield, and quality. However, plants deploy defense mechanisms, including the synthesis of SMs, to mitigate the adverse effects of these stressors. SMs, comprising various chemical classes such as phenolics, alkaloids, terpenoids, and flavonoids, play pivotal roles in plant adaptation, defense, and stress response mechanisms. These organic compounds are synthesized via complex biochemical pathways and contribute to plant growth regulation, reproduction, and resilience to environmental challenges. This section discusses SMs’ key roles in stress mitigation in various crops, with a focus on antioxidant activity, osmotic regulation, membrane stabilization, and involvement in hormonal and signaling networks. Flavonoids and anthocyanins have been found to accumulate in response to drought and salinity stress, boosting plant resistance by scavenging ROS and lowering oxidative damage. The buildup of soluble sugars, organic osmolytes, and flavonoids is critical for maintaining cellular turgor pressure under salt and drought stress. The buildup of phenolic acids, such as chlorogenic acid, has been associated with improved membrane integrity during drought stress, limiting disruption of cellular processes and preserving plant vigor. The synergy of phytohormones such as ABA, SA, and SM, as well as flavonoids, influences plants’ ability to regulate the balance of primary and SM synthesis. Anthocyanins under heat and salinity stress have been associated with increased photosynthetic efficiency by inhibiting photosynthetic machinery. Biotechnological interventions have emerged as powerful tools for enhancing the resilience of forage crops to abiotic stress. Various omics approaches offer comprehensive insights into the molecular mechanisms underlying stress responses and crop resilience. Genome editing technologies, particularly CRISPR-Cas9, provide precise means for engineering pathway alterations that hold significant potential for enhancing the composition and digestibility of forage crops, thereby improving their nutritional value and suitability for livestock feed and addressing the increasing demand for sustainable agricultural resources.

### 5.2 Future prospects

Bottlenecks in improving the production of SMs in plants are majorly due to the intricacies of biosynthetic pathways, the scarcity of genomic resources for non-model plants, and trade-offs between metabolite production and crop yield. Added complications include variable environmental conditions that make *in vitro* results difficult to apply in *in vivo* and/or in the field and regulations that arise over ethical concerns pertaining to genetically modified crops. Advancement in future lines of work related to SM enhancement in plants must be through multi-omics integration, as this would help in systems-level understanding of the biosynthesis and identification of novel pathways. Synthetic biology can further engineer multiple stress tolerance traits with novel biosynthetic pathways leading to diverse metabolite production. Post-harvest, it must balance carbon flow such that it maximizes yield in metabolite production as well as the scale of growth improvement. Continued research and technological advancements in these areas are crucial for addressing the challenges posed by changing environmental conditions and ensuring the availability of high-quality forage resources for sustainable agriculture. By unraveling the intricate interplay between abiotic stress and SM production, researchers can pave the way for innovative approaches to enhance crop resilience and sustainability, ultimately contributing to global food security and agricultural sustainability in the face of ongoing climate change and environmental pressures.

Several questions remain unanswered to pose challenges to research in this area. One such question is how the entire biosynthetic pathways of major SMs can be properly identified, charted, and controlled, especially in non-model forage and fodder crops that have inherently intricate metabolic networks. A further essential gap exists in the effective generation of detailed omics resources—such as genomic, transcriptomic, proteomic, and metabolomic data sets—for these non-model organisms. Furthermore, it is unknown which approaches and strategies can effectively navigate the balance between greater SM synthesis and preserving or enhancing crop yield under stress. Lastly, one of the greatest challenges is reliably translating *in vitro* results on SM biosynthesis and stress tolerance into whole-plant (*in vivo*) and field environments, where the plant experiences fluctuating and unpredictable environmental conditions.
