# Supplementary material for: Abiotic stress responses in forage crops and grasses: the role of secondary metabolites and biotechnological interventions
Source: Front Plant Sci. 2025 Jun 3;16:1542519. doi: 10.3389/fpls.2025.1542519 (PMC12188474; doi:10.3389/fpls.2025.1542519)
Supplement: Supplementary file 1 [file Table1.docx]

**Supplementary Table** **1:** Comparative SM pathway with gene orthologs considering all SMs that have been curated for or are known is provided below

| **Sl. No.** | **Pathway Name** | **Type of Pathway** | **Pathway genes** | ***Zea mays*** | ***Trifolium pratense*** | ***Sorghum bicolor*** | ***Saccharum spontaneum*** | ***Hordeum vulgare*** | ***Brassica napus*** | ***Brassica rapa*** | ***Beta vulgaris*** |
| --- | --- | --- | --- | --- | --- | --- | --- | --- | --- | --- | --- |
| 1 | (3E)-4,8-dimethylnona-1,3,7-triene biosynthesis I | Secondary Metabolite Biosynthesis | TPS1  CYP82G1 | (3E)-4,8-dimethylnona-1,3,7-triene biosynthesis I | (3E)-4,8-dimethylnona-1,3,7-triene biosynthesis I | (3E)-4,8-dimethylnona-1,3,7-triene biosynthesis I | (3E)-4,8-dimethylnona-1,3,7-triene biosynthesis I | - | (3E)-4,8-dimethylnona-1,3,7-triene biosynthesis I | (3E)-4,8-dimethylnona-1,3,7-triene biosynthesis I | (3E)-4,8-dimethylnona-1,3,7-triene biosynthesis I |
| 2 | 2'-deoxymugineic acid phytosiderophore biosynthesis | Secondary Metabolite Biosynthesis | pOS-SAMS2  OsNAS1  OsNAS2  OsNAS3  hvnas1  OsNAAT1  naatA  naatB  OsDMAS1  hvDMAS1  TaDMAS1  ZmDMAS1 | 2'-deoxymugineic acid phytosiderophore biosynthesis | - | 2'-deoxymugineic acid phytosiderophore biosynthesis | 2'-deoxymugineic acid phytosiderophore biosynthesis | 2'-deoxymugineic acid phytosiderophore biosynthesis | - | - | - |
| 3 | 3,8-divinyl-chlorophyllide a biosynthesis I (aerobic, light-dependent) | Tetrapyrrole Biosynthesis  Secondary Metabolite Biosynthesis | PPXII  PPOX  Cre09.g396300  UROD  Zm00001d029074  CHLI  CHLH  CHLD  AT4G18480  GUN5  CHLD  Cre07.g325500  l13  GRMZM2G043453_P01  oy1  CHLM  HEMF1  Cre02.g085450  Zm00001d002358  CHL27 | 3,8-divinyl-chlorophyllide a biosynthesis I (aerobic, light-dependent) | 3,8-divinyl-chlorophyllide a biosynthesis I (aerobic, light-dependent) | 3,8-divinyl-chlorophyllide a biosynthesis I (aerobic, light-dependent) | 3,8-divinyl-chlorophyllide a biosynthesis I (aerobic, light-dependent) | 3,8-divinyl-chlorophyllide a biosynthesis I (aerobic, light-dependent) | 3,8-divinyl-chlorophyllide a biosynthesis I (aerobic, light-dependent) | 3,8-divinyl-chlorophyllide a biosynthesis I (aerobic, light-dependent) | 3,8-divinyl-chlorophyllide a biosynthesis I (aerobic, light-dependent) |
| 4 | ABA biosynthesis | Secondary Metabolite Biosynthesis  Hormone, Neurotransmitter, and Signaling Molecule Biosynthesis | ABA2  AAO3  Zm00001d034385  NCED2  NCED6  NCED9  NCED3  Zm00001d033222 | ABA biosynthesis | ABA biosynthesis | ABA biosynthesis | ABA biosynthesis | ABA biosynthesis | ABA biosynthesis | ABA biosynthesis | ABA biosynthesis |
| 5 | Acetaldehyde biosynthesis I | Secondary Metabolite Biosynthesis | ADH2  ADH1  adh  ADH  ADH1 | Acetaldehyde biosynthesis I | Acetaldehyde biosynthesis I | Acetaldehyde biosynthesis I | Acetaldehyde biosynthesis I | Acetaldehyde biosynthesis I | Acetaldehyde biosynthesis I | Acetaldehyde biosynthesis I | Acetaldehyde biosynthesis I |
| 6 | All-trans-farnesol biosynthesis | Secondary Metabolite Biosynthesis | TPS1 | - | - | - | all-trans-farnesol biosynthesis | - | - | - | - |
| 7 | Anthocyanin biosynthesis | Secondary Metabolite Biosynthesis  Aromatic Compound Biosynthesis | pGUT8  UGT78D2  Glyma.07G183200  Glyma.12G163432  Glyma.12G163366  Glyma.08G066800  Glyma.15G058800  Glyma.07G183400  Glyma.07G183300  Glyma.13G255800  Glyma.U001500  Glyma.06G252566  Glyma.05G181100  Glyma.06G239600  Glyma.06G239500  Glyma.03G032800  Glyma.03G032700  Glyma.03G032500  Glyma.03G032600 | Anthocyanin biosynthesis | Anthocyanin biosynthesis | Anthocyanin biosynthesis | Anthocyanin biosynthesis | Anthocyanin biosynthesis | Anthocyanin biosynthesis | Anthocyanin biosynthesis | Anthocyanin biosynthesis |
| 8 | Apigeninidin 5-O-glucoside biosynthesis | Secondary Metabolite Biosynthesis  Aromatic Compound Biosynthesis | DFR/FNR  A1  UGT5 | Apigeninidin 5-O-glucoside biosynthesis | Apigeninidin 5-O-glucoside biosynthesis | Apigeninidin 5-O-glucoside biosynthesis | Apigeninidin 5-O-glucoside biosynthesis | Apigeninidin 5-O-glucoside biosynthesis | Apigeninidin 5-O-glucoside biosynthesis | Apigeninidin 5-O-glucoside biosynthesis | Apigeninidin 5-O-glucoside biosynthesis |
| 9 | Bergamotene biosynthesis I | Secondary Metabolite Biosynthesis | LaBERS  TPS10 | - | - | - | - | - | - | - | - |
| 10 | β-carotene biosynthesis (engineered) | Superpathways  Secondary Metabolite Biosynthesis | LCY1  PSY1  PSY | - | - | - | - | - | - | - | - |
| 11 | β-caryophyllene biosynthesis | Secondary Metabolite Biosynthesis | tps23  TPS23  QHS1  bcp  tps23  OsTPS3  AT5G23960 | β-caryophyllene biosynthesis | β-caryophyllene biosynthesis | β-caryophyllene biosynthesis | β-caryophyllene biosynthesis | β-caryophyllene biosynthesis | β-caryophyllene biosynthesis | β-caryophyllene biosynthesis | - |
| 12 | Cycloartenol biosynthesis | Secondary Metabolite Biosynthesis  Fatty Acid and Lipid Biosynthesis | OSCPNX1  cs1  RcCAS  KcCAS  ACX  CAS1  LOC_Os02g04710 | Cycloartenol biosynthesis | Cycloartenol biosynthesis | Cycloartenol biosynthesis | Cycloartenol biosynthesis | Cycloartenol biosynthesis | Cycloartenol biosynthesis | Cycloartenol biosynthesis | Cycloartenol biosynthesis |
| 13 | DIBOA-glucoside biosynthesis | Secondary Metabolite Biosynthesis | Bx3  Bx2  Bx5  Bx4  Bx1  Igl  Bx8 | DIBOA-glucoside biosynthesis | DIBOA-glucoside biosynthesis | DIBOA-glucoside biosynthesis | DIBOA-glucoside biosynthesis | DIBOA-glucoside biosynthesis | - | - | DIBOA-glucoside biosynthesis |
| 14 | DIMBOA-glucoside biosynthesis | Secondary Metabolite Biosynthesis | Bx7  bx7  Bx6 | DIMBOA-glucoside biosynthesis | - | DIMBOA-glucoside biosynthesis | DIMBOA-glucoside biosynthesis | DIMBOA-glucoside biosynthesis | - | - | DIMBOA-glucoside biosynthesis |
| 15 | Dolabralexins biosynthesis | Secondary Metabolite Biosynthesis | CYP71Z16  CYP71Z18  KSL4 | Dolabralexins biosynthesis | - | Dolabralexins biosynthesis | Dolabralexins biosynthesis | - | - | - | - |
| 16 | Ent-kaurene biosynthesis I | Secondary Metabolite Biosynthesis  Hormone, Neurotransmitter, and Signaling Molecule Biosynthesis | Cyc2  CPS1  CPS  cpps2  Zm00001d032961  Zm00001eb167120  KSB  KS1  TPS1 | Ent-kaurene biosynthesis I | Ent-kaurene biosynthesis I | Ent-kaurene biosynthesis I | Ent-kaurene biosynthesis I | Ent-kaurene biosynthesis I | Ent-kaurene biosynthesis I | Ent-kaurene biosynthesis I | Ent-kaurene biosynthesis I |
| 17 | Epoxysqualene biosynthesis | Secondary Metabolite Biosynthesis  Fatty Acid and Lipid Biosynthesis | SE  SQE1  SQE3  SQE2  PSS  SQS2  SQS1 | Epoxysqualene biosynthesis | Epoxysqualene biosynthesis | Epoxysqualene biosynthesis | Epoxysqualene biosynthesis | Epoxysqualene biosynthesis | Epoxysqualene biosynthesis | Epoxysqualene biosynthesis | Epoxysqualene biosynthesis |
| 18 | Gibberellin A_12_ biosynthesis | Secondary Metabolite Biosynthesis  Hormone, Neurotransmitter, and Signaling Molecule Biosynthesis | KO1  GA3  KAO2  KAO1  KAO1  KAO2  Zm00001d045563 | Gibberellin A_12_ biosynthesis | Gibberellin A_12_ biosynthesis | Gibberellin A_12_ biosynthesis | Gibberellin A_12_ biosynthesis | Gibberellin A_12_ biosynthesis | Gibberellin A_12_ biosynthesis | Gibberellin A_12_ biosynthesis | Gibberellin A_12_ biosynthesis |
| 19 | Geraniol and geranial biosynthesis | Secondary Metabolite Biosynthesis | GES  PfTps-PL  PcTps-EK  PcTps-C  GES  TPS1  GEDH1  CAD1 | Geraniol and geranial biosynthesis | Geraniol and geranial biosynthesis | Geraniol and geranial biosynthesis | Geraniol and geranial biosynthesis | Geraniol and geranial biosynthesis | - | - | Geraniol and geranial biosynthesis |
| 20 | Gibberellin biosynthesis III (early C-13 hydroxylation) | Secondary Metabolite Biosynthesis  Hormone, Neurotransmitter, and Signaling Molecule Biosynthesis | GA20ox2  GA20ox2  GA20ox1D  SD1  GA20ox  GA20ox3  GA20ox1  GA20ox  AT1G80330  GA3ox2  GA3ox2-1  GA3ox1  GA3ox1 | Gibberellin biosynthesis III (early C-13 hydroxylation) | Gibberellin biosynthesis III (early C-13 hydroxylation) | Gibberellin biosynthesis III (early C-13 hydroxylation) | Gibberellin biosynthesis III (early C-13 hydroxylation) | Gibberellin biosynthesis III (early C-13 hydroxylation) | Gibberellin biosynthesis III (early C-13 hydroxylation) | Gibberellin biosynthesis III (early C-13 hydroxylation) | Gibberellin biosynthesis III (early C-13 hydroxylation) |
| 21 | Hydroxycinnamic acid tyramine amides biosynthesis | Secondary Metabolite Biosynthesis | TYDC1  TYDC2  THT | Hydroxycinnamic acid tyramine amides biosynthesis | - | - | - | - | - | - | - |
| 22 | Jasmonic acid biosynthesis | Secondary Metabolite Biosynthesis  Hormone, Neurotransmitter, and Signaling Molecule Biosynthesis | AT3G25770  OPR3  Zm00001d032049  opr8  CYP74A1  Zm00001d048021  LOX2  LOX3  LOX6  LOX4  LOX3  LOX2  Zm00001d053675  Zm00001d003533  OPCL1  AIM1  KAT2  ACX1  ACX5 | Jasmonic acid biosynthesis | Jasmonic acid biosynthesis | Jasmonic acid biosynthesis | Jasmonic acid biosynthesis | Jasmonic acid biosynthesis | Jasmonic acid biosynthesis | Jasmonic acid biosynthesis | Jasmonic acid biosynthesis |
| 23 | Kauralexin biosynthesis | Superpathway  Secondary Metabolite Biosynthesis | TPS1  cpps2  Zm00001d032961  Zm00001eb167120 | - | - | - | - | - | - | - | - |
| 24 | Luteolinidin 5-O-glucoside biosynthesis | Secondary Metabolite Biosynthesis  Aromatic Compound Biosynthesis | DFR/FNR  DFR  A1  UGT5 | Luteolinidin 5-O-glucoside biosynthesis | Luteolinidin 5-O-glucoside biosynthesis | Luteolinidin 5-O-glucoside biosynthesis | Luteolinidin 5-O-glucoside biosynthesis | Luteolinidin 5-O-glucoside biosynthesis | Luteolinidin 5-O-glucoside biosynthesis | Luteolinidin 5-O-glucoside biosynthesis | Luteolinidin 5-O-glucoside biosynthesis |
| 25 | Phytosterol biosynthesis (plants) | Secondary Metabolite Biosynthesis  Fatty Acid and Lipid Biosynthesis | SMT1  CPI1  FK  HYD1  DWF5  CYP710A1  SMT2-2  SMT2-1  SMT2  SMT3  CYP710A2  AT3BETAHSD/D2  AT3BETAHSD/D1  DWF7  CYP51G1  OBT14DM  AT1G07420  DWF1 | Phytosterol biosynthesis (plants) | Phytosterol biosynthesis (plants) | Phytosterol biosynthesis (plants) | Phytosterol biosynthesis (plants) | Phytosterol biosynthesis (plants) | Phytosterol biosynthesis (plants) | Phytosterol biosynthesis (plants) | Phytosterol biosynthesis (plants) |
|  |  |  |  |  |  |  |  |  |  |  |  |
| 26 | Superpathway of benzoxazinoid glucosides biosynthesis | Superpathways  Secondary Metabolite Biosynthesis | Bx3  Bx2  Bx5  Bx4  Bx1  Igl  Bx8  Bx7  bx7  Bx6 | - | - | - | - | - | - | - | - |
| 27 | Trans-lycopene biosynthesis II (oxygenic phototrophs and green sulfur bacteria) | Secondary Metabolite Biosynthesis | Z-ISO  Z-ISO  PSY  PSY1  CRTISO  CA08g10750  ZDS  ZDS  PDS | Trans-lycopene biosynthesis II (oxygenic phototrophs and green sulfur bacteria) | Trans-lycopene biosynthesis II (oxygenic phototrophs and green sulfur bacteria) | Trans-lycopene biosynthesis II (oxygenic phototrophs and green sulfur bacteria) | Trans-lycopene biosynthesis II (oxygenic phototrophs and green sulfur bacteria) | Trans-lycopene biosynthesis II (oxygenic phototrophs and green sulfur bacteria) | Trans-lycopene biosynthesis II (oxygenic phototrophs and green sulfur bacteria) | Trans-lycopene biosynthesis II (oxygenic phototrophs and green sulfur bacteria) | Trans-lycopene biosynthesis II (oxygenic phototrophs and green sulfur bacteria) |
| 28 | Zealexin biosynthesis | Secondary Metabolite Biosynthesis | TPS10  TPS6  CYP71Z18 | Zealexin biosynthesis | - | - | Zealexin biosynthesis | - | - | - | - |

‘-’ represents not available/not found in the respective crops

Secondary metabolites those which have been curated for or are known are represented in the above table

**Source:** <https://pmn.plantcyc.org/group?id=pmn.plantcyc.org-7711-3920509542>
